# Octopus-Inspired Underwater Soft Robotic Gripper with Crawling and Swimming Capabilities

**DOI:** 10.34133/research.0456

**Published:** 2024-08-28

**Authors:** Mingxin Wu, Waqar Hussain Afridi, Jiaxi Wu, Rahdar Hussain Afridi, Kaiwei Wang, Xingwen Zheng, Chen Wang, Guangming Xie

**Affiliations:** ^1^ State Key Laboratory for Turbulence and Complex Systems, Intelligent Biomimetic Design Lab, College of Engineering, Peking University, Beijing 100871, China.; ^2^ National Engineering Research Center of Software Engineering, Peking University, Beijing 100871, China.; ^3^ Institute of Ocean Research, Peking University, Beijing 100871, China.

## Abstract

Can a robotic gripper only operate when attached to a robotic arm? The application space of the traditional gripper is limited by the robotic arm. Giving robot grippers the ability to move will expand their range of applications. Inspired by rich behavioral repertoire observed in octopus, we implement an integrated multifunctional soft robotic gripper with 6 independently controlled Arms. It can execute 8 different gripping actions for different objects, such as irregular rigid/soft objects, elongated objects with arbitrary orientation, and plane/curved objects with larger sizes than the grippers. Moreover, the soft gripper can realize omnidirectional crawling and swimming by itself. The soft gripper can perform highly integrated tasks of releasing, crawling, swimming, grasping, and retrieving objects in a confined underwater environment. Experimental results demonstrate that the integrated capabilities of multimodal adaptive grasping and omnidirectional motions enable dexterous manipulations that traditional robotic arms cannot achieve. The soft gripper may apply to highly integrated and labor-intensive tasks in unstructured underwater environments, including ocean litter collecting, capture fishery, and archeological exploration.

## Introduction

The ocean, the cradle of life, provides essential resources and facilities for humans. Plastic waste is a severe environmental problem that takes hundreds of years to degrade when it sinks to the seafloor and can cause human health problems through the global carbon cycle [1,2]. Therefore, it is necessary to clean them up to avoid irreversible environmental damage. At the same time, the abundant seafood in the ocean plays a critical role as a primary source of protein for humanity [3]. Furthermore, the ocean serves as one of the world’s primary channels for transportation and trade, and exploring sunken cargo ships can be of valuable economic and historical significance. Tasks such as the cleanup of marine solid pollutants, fishing, and underwater archaeology are labor-intensive. Therefore, there is an urgent need for underwater robots to assist or even replace humans [4] in carrying out these tasks related to marine protection and development.

The robotic gripper typically serves as the end effector for underwater robotic manipulations during the above tasks. Expanding its applications to adaptively grasp a variety of objects in unstructured underwater environments is a substantial challenge. In nature, cephalopods, such as octopuses, display the ability for underwater multimodal motion [5] and adaptive grasping [6–8]. These unparalleled characteristics make octopuses one of the most popular animal models guiding the design of next-generation underwater grippers. Several robotic prototypes have already been inspired by the octopus and perform various functions. These include robotic grippers [9–11] and suckers [12,13] for adaptive grasping, underwater crawling robots [14,15], swimming robots [16–20], and robots designed for underwater manipulation and movement [21,22]. However, their grasping and movement capabilities rely on different systems. Moreover, underwater robots carrying grippers are usually large and cannot enter narrow spaces. The application scenarios of underwater robots may be significantly broadened by integrating these diverse functions—adaptive grasping, omnidirectional crawling, and 3D swimming—into the robotic gripper of an underwater robot’s robotic arm. Although some robotic grippers for underwater use have already been developed, such as forked metal or plastic jaws [23,24], sucker grippers [10], rotary-actuated folding polyhedrons for midwater investigation [25], soft hydraulic actuators [26,27], and soft manipulators for efficient grasping [28], these grippers can only be fixed on the robotic arm, and the use space of the grippers is also limited by the robotic arm. As a result, robotic grippers cannot grasp objects in confined underwater spaces (such as coral reef formations, rock crevices, and inside shipwreck hulls) where the robot or robotic arm cannot reach them. Additionally, soft grippers on underwater robots ensure compliant interaction with objects for adaptive grasping due to their inherent compliance contrary to rigid grippers. Nevertheless, the gap between the fingers of soft-fingered grippers [29,30] and the target object cannot be eliminated, which may inhibit grasping stability [31]. Vacuum-driven, no fingered grippers based on granular jamming [32,33] or the origami design [34] can adapt to objects of any shape, but their grasping mode is singular and limited to objects smaller than the size of the gripper. The application of suction-based grippers [35–37] is often limited to objects with flat or nearly flat surfaces. In addressing the variety of underwater grasping objects, particularly underwater litter collections, the grasping capability of existing soft grippers proves to be limited.

**Fig. 1.**
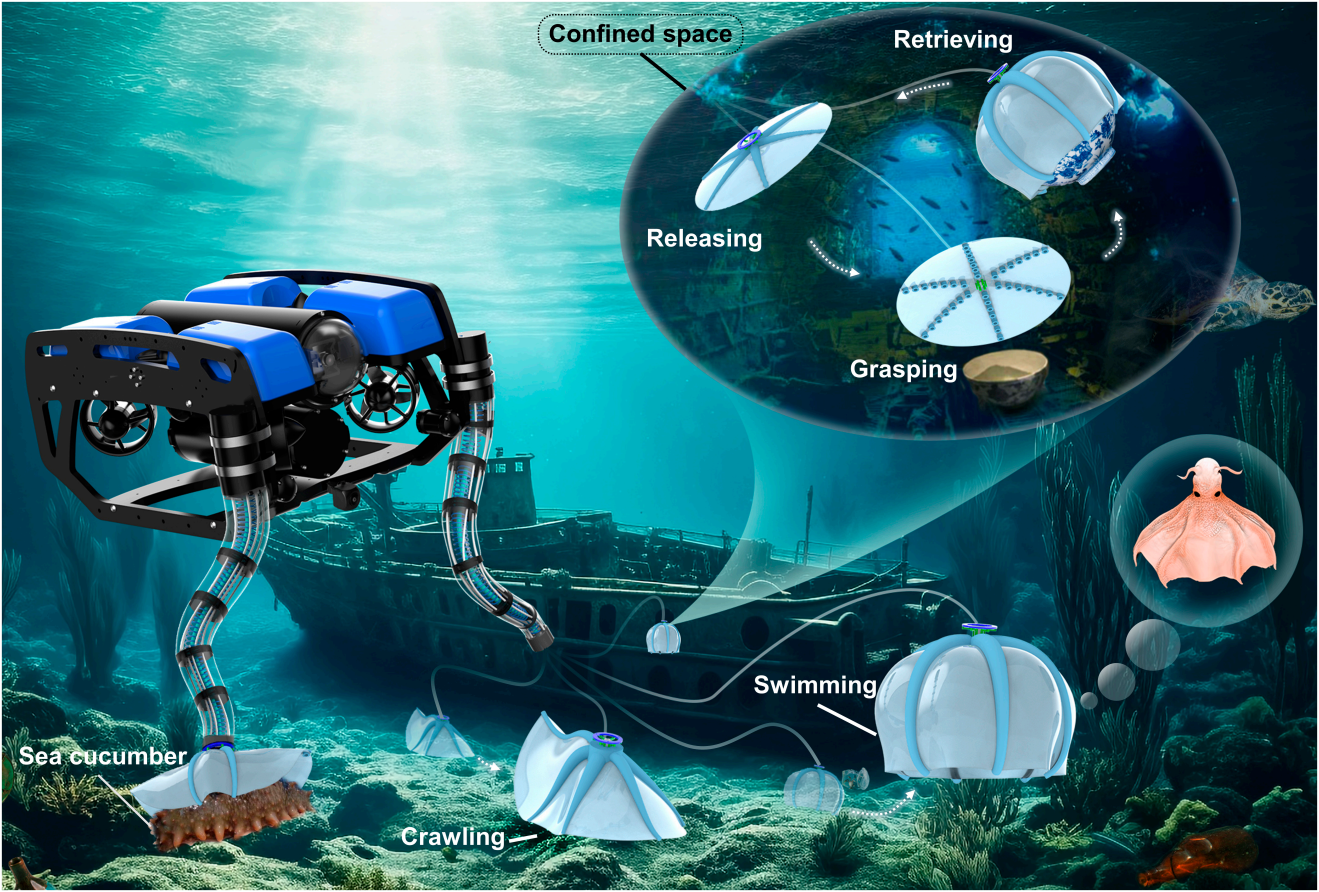
A depiction of the mission profile and design of the octopus-inspired underwater soft gripper system. The gripper integrates adaptive grasping, omnidirectional crawling, and 3D swimming capabilities.

Inspired by the synergy of octopus arms, which are adept at adaptive grasping and underwater movement, we have developed a soft gripper with 6 Arms (where “Arm” denotes a single component of the gripper). The 6 Arms engage in harmonious operation to equip the gripper with 2 distinct states: grasping and movement. When attached to a robotic arm, the gripper operates in a multimodal adaptive grasping mode. However, when detached from the robotic arm, it can perform underwater movements and grasping independently. The harmonious operation of the multiple Arms integrates 3 key functionalities into the soft gripper: grasping, omnidirectional crawling, and 3-dimensional (3D) swimming (as depicted in Fig. 1). In the grasping state, the system can generate 8 different grasping modes based on the object being held. These modes include the following: (a) Seven closing–opening grasping actions enabled by Arms: The 6 Arms are driven in coordination by pumps while bending 2 or 4 Arms in arbitrary postures, resulting in 6 grasping modes. An additional mode is achieved when all 6 Arms are simultaneously actuated. (b) One extra grasping action enabled by suckers: The sucker system’s grasp modes are uniformly driven by positive and negative-pressure control of the pump, enabling pre-adhesion, enhanced negative-pressure adhesion, and positive-pressure release. Integrating multiple grasping modes enables adaptive grasping of irregular rigid/flexible objects, elongated objects placed in any direction, and plane/curved objects larger than the gripper’s diameter. In the movement state, actuating any 2 Arms separated by an unactuated Arm allows the gripper to perform underwater crawling and omnidirectional movement. By bending all Arms, the gripper achieves both vertical swimming and precise control over the direction of descent, enabling 3D swimming. Furthermore, this gripper can execute advanced operations such as wired releasing, crawling, swimming, grasping, and retrieving objects within confined underwater spaces. The gripper integrates multiple functions, providing a reliable solution for adaptive grasping and exploration in confined spaces by combining it with the robotic arm. This significantly expands the application range of underwater grippers, making it suitable for highly integrated and labor-intensive tasks in unstructured underwater environments, including ocean litter collection, capture fisheries, and archaeological exploration.

## Results

### Mechanisms and characterizations of the soft gripper

**Fig. 2.**
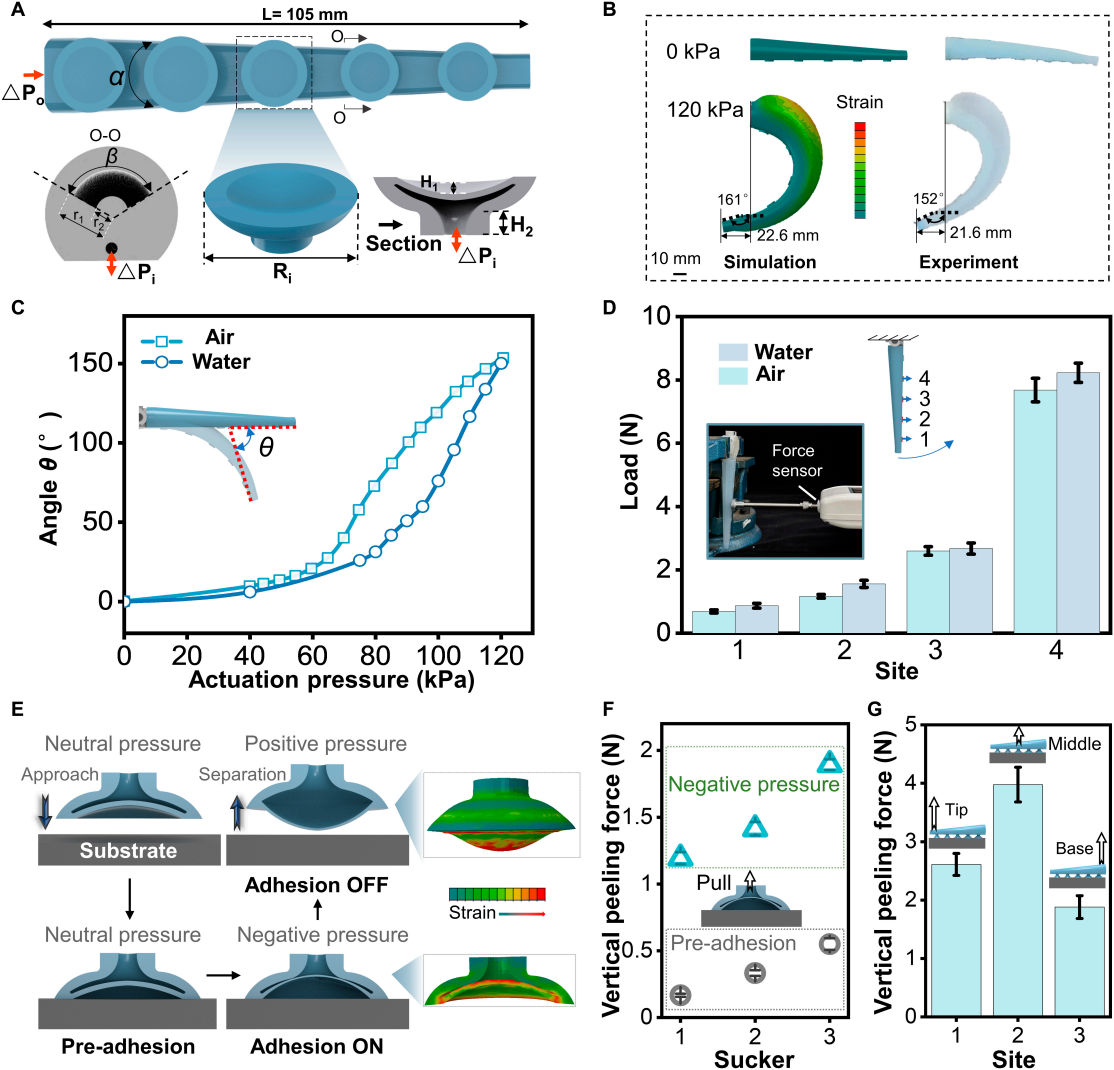
Soft Arm design and the principle of actuation. (A) Illustration of the soft Arm. The soft gripper consists of 6 Arms with 5 suckers on each Arm. (B) Simulation of the finite-element model (left) and experimental result of a soft Arm (right) under an applied pressure of 120 kPa. (C) Bending angle of the Arm under air and water actuation. (D) The load capacity test corresponds to each sucker point on the Arm driven by 2 fluids (water or air). Error bars show standard deviation from 4 tests. (E) Schematic showing the test procedure for underwater adhesive switch characteristics and simulation of the sucker. (F) Changes in the pre-adhesion and negative-pressure force of suckers of different diameters peeled off in the vertical direction. Error bars show standard deviation from 4 tests. (G) Suction during vertical peeling of an Arm adhered to a flat substrate from different positions. Error bars show standard deviation from 4 tests.

The soft gripper (Fig. 1) consists of Arms, suckers, and a ventral membrane. The tapered Arm length (*L*) is 105 mm, the taper angle (α) is 5.7°, and the bending is achieved by ***△**P_o_* driving (Fig. 2A). The internal chamber of the Arm is circular. The figure shows the cross-section of the Arm’s inner chamber at *L* = 40 mm, where the swept arch-shaped *β* = 120°, the inner radius *r*_1_ = 1.75 mm, and the outer radius *r*_2_ = 3.37 mm. Five suckers are distributed along each Arm, driven by *△Pi*. From right to left, the diameters of the suckers are *R*_1_ = 6 mm *2, *R*_2_ = 7 mm (center), and *R*_3_ = 8 mm *2. The height of each sucker remains consistent, with *H_1_* = 1.5 mm and *H_2_* = 2 mm. We further developed a finite element model to quantitatively predict the deformation and bending angle of the Arm after actuation (see the “Finite element method simulations” section in Materials and Methods). The finite element model predicts that the Arm under the actuation of 120 kPa gives the bending angles *θ*_max_ of 161°, the end exceeds the fixed position of the gripper by 22.6 mm in the vertical direction, and the deviation from the experimental results is only 5.6% and 4.4% (Fig. 2B). The finite element model has a high degree of agreement with the bending shape of the experimental results, which provides a reference for the design of our single Arm and gripper. To explore the feasibility of utilizing the Arm in both air and water environments, we conducted a comparative study on the bending state of the Arm and its gripping force output under different media drives (Fig. 2C and D). Due to the higher density of air than water, the response time was significantly faster. The maximum bending angles *θ*_max_ of the 2 driving methods were similar (Fig. 2C). The analytical model for the Arm design can be found in Fig. S1. Moreover, the Arm is highly flexible (Fig. S2) and bends continuously at the point of contact with an obstacle, forming a joint (Movie S1). This characteristic provides a foundation for adaptive gripping by the Arm. In addition, comparing the force generated by the gripper driven by the 2 fluids under 140 kPa (Fig. 2D), it is found that there is a gradually decreasing trend in holding force output under both driving fluids from the fixed end to the tip of the Arm. This effect should be the focus of future efforts, designing variable stiffness [38–40] to enable the arm’s end to output greater holding force after gripping, ensuring gripping stability.

The schematic of Fig. 2E illustrates the sequence of testing for a sucker with negative and positive pressure. First, the sucker approaches the substrate (acrylic sheet) until it touches it, which creates a pre-adhesion. Next, negative pressure was applied to activate the sucker (Adhesion ON). The adhesion was held for 1 s and subsequently pulled from the substrate until separation occurred. Finally, rapid separation can be achieved by applying positive pressure against the substrate. The iterative design of the sucker was achieved by establishing a finite element model for prior simulations (see the “Finite element method simulations” section in Materials and Methods). In addition, the changes in the pre-adhesion and negative-pressure force of the suckers with diameters *R*_1_, *R*_2_, and *R*_3_ peeled off in the vertical direction were compared through experiments. The results show that the larger the diameter of the sucker, the greater the initial pre-adhesion and the greater the adhesion driven by negative pressure. The adhesion switching ratios of the 3 suckers were over 7.16×, 4.24×, and 3.44× from pre-adhesion to Adhesion ON (Fig. 2F). The above experiments demonstrate the superiority of the sucker design, which shows that pre-adhesion may improve the success rate of grasping, producing negative pressure to enhance adhesion and positive pressure to release objects quickly. The actual operation diagram of the sucker is shown in Fig. S3 and Movie S2. After all the suckers were integrated into the Arm, a single Arm adhered to a flat substrate to test the variation of tensile force by vertical peeling from different positions (Fig. 2G and Movie S3). The results showed that the maximum adhesion occurred when the tensile force was applied in the middle of the Arm, and the substrate was separated from the sucker instantly, causing a sharp drop in adhesion during the peeling process. When a tensile force was applied at the tip or base of the Arm, the suckers were detached from the substrate one by one, which did not lead to a sharp drop in adhesion (Fig. S4). The force that separates the suckers from the arm is much greater than the suction force of the suckers, so the suckers will not fall off during operation (Fig. S5). This shows that each sucker can form independent compartments and achieve redundant adhesion just like its biological counterpart.

### Actuation principles

**Fig. 3.**
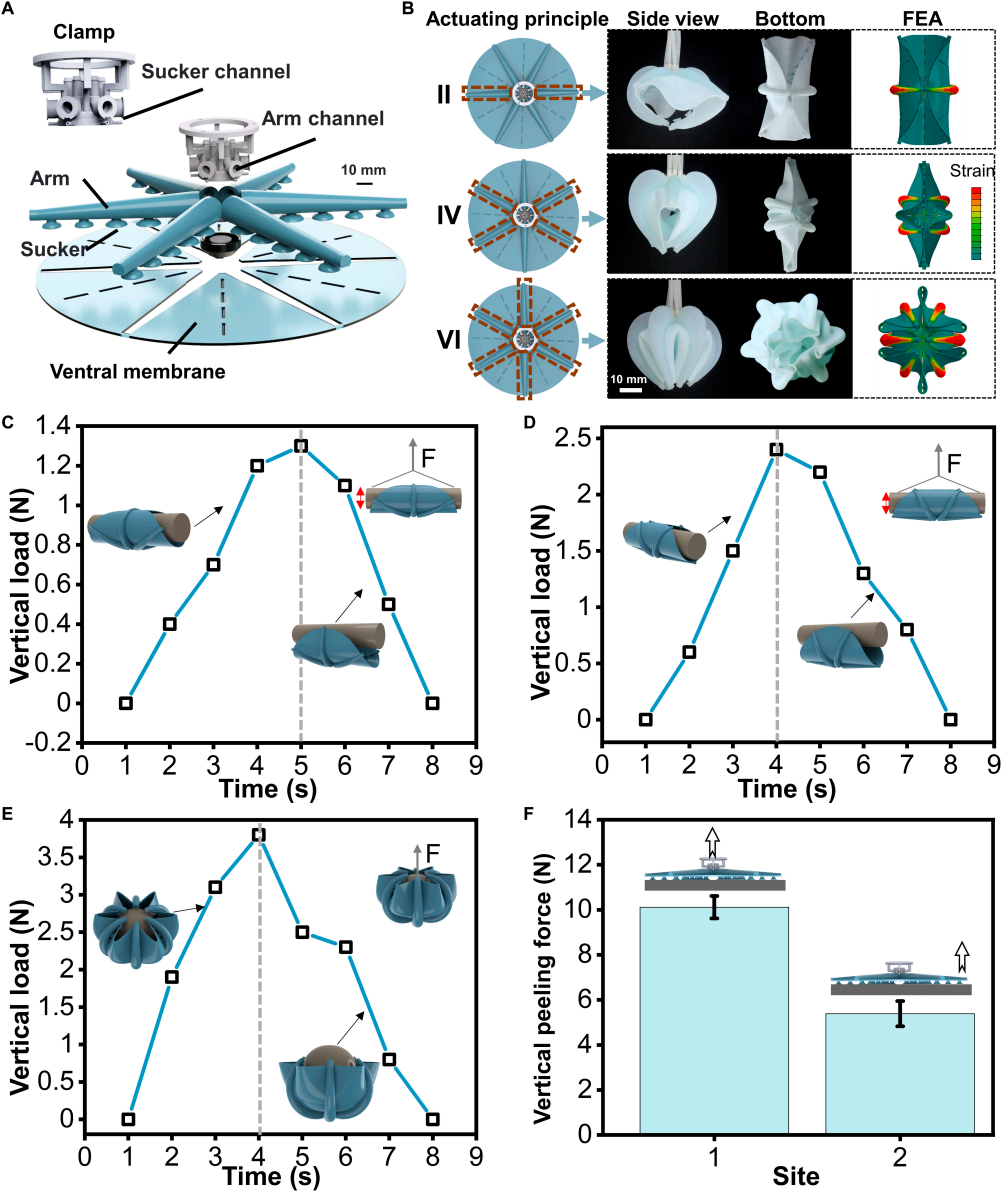
Performance characterization of the soft gripper. (A) The 2-dimensional (2D) layered design of the soft gripper (the design details of the clamp can be found in Fig. S8). (B) Three actuation principles of the soft gripper and the corresponding simulation and experimental results. (C to E) Vertical load capacity test for actuation principles II (C), IV (D), and VI (E) with a soft gripper gripping a 90-mm-diameter cylinder at 140-kPa pressure. During these tests, the soft gripper was fixed to the testbed, and the horizontal cylinder was positioned at the center of the gripper. Then, the soft Arms were actuated to the predefined pressures. The linear stage pulls the cylinder at a fixed velocity (12.5 mm s^−1^ ) until the cylinder separates from the soft gripper. (F) The suction is generated by the soft gripper in the suction mode when the vertical peeling position is at the center and the edge, respectively. Error bars show standard deviation from 4 tests.

We used a simple and scalable method to fabricate and assemble the soft gripper (see the “Fabrication and assembly of the soft gripper” section in Materials and Methods; Figs. S6 and S7). Each gripper consists of 6 Arms, 30 suckers, and the ventral membrane, while the gripper system is assembled to the robotic arm by a clamp (Fig. 3A and Fig. S8). The suction mode is achieved by the unified control of the suckers on all Arms through the sucker channels on the clamp. The grasping mode is achieved by controlling each Arm individually through the Arm channel. A ventral membrane similar to that of an octopus (*Stauroteuthis syrtensis*) assists the gripper in underwater movement. Besides, it helps create a more secure and stable grip by conforming to the shape of the object being grasped, especially for irregularly shaped objects or when grasping multiple objects simultaneously. Four rectangular gaps on each ventral membrane allow water to pass through at a faster-falling speed and a more stable landing point when released underwater (Fig. S9). The soft gripper has 3 kinds of actuating principles: actuating 2 Arms symmetrically (principle II), actuating 4 Arms symmetrically (principle IV), and actuating 6 Arms simultaneously (principle VI) (Table 1). Principle II of arbitrarily symmetrical 2-Arm actuation can derive 3 grasping modes, and both the side view and the bottom view of this principle are shown in Fig. 3B. Correspondingly, principle IV of arbitrarily symmetrical 4-Arm actuation can also derive 3 grasping modes (Fig. 3B). The principle of simultaneous actuation of 6 Arms (principle VI) has only one grasping mode (Fig. 3B). The finite element model was established to predict the various grasping principles of the soft gripper. The results show that there was relatively good correspondence. We next characterized the performance of the soft gripper. To evaluate the load capacity of the gripper, we tested actuation principles II and IV (at 140 kPa pressure) to hold a cylinder and actuation principle VI to grasp a sphere while measuring the grasping force using an electronic dynamometer (Fig. S10). Figure 3C to E shows the whole process of the object (90-mm-diameter cylinder) from being grasped to wholly detached from the soft gripper. The dashed lines are the position of maximum gripping force. The results showed that the maximum grasping force of the soft gripper under the 3 grasping principles was 1.3, 2.4, and 3.8 N, respectively (Fig. 3C to E). More test results for cylinders and spheres of different diameters can be found in Figs. S10 and S11. In addition, vertical peeling was tested in 2 different ways (Fig. 3F) with the planar base bent over the soft gripper in suction mode (Fig. S12 and Movie S4), and the peeling from the center showed a maximum bearing capacity of 10.5 N. Therefore, the grasping force output by the soft gripper can accomplish most of the grasping of underwater litter [41]. The grasping force of the soft gripper could be increased by using stiffer elastomers in the Arms and applying higher pressure to reach the requirements of various tasks.

**Table 1.** Derived grasping modes and application scenarios corresponding to different actuation principles of the soft gripper

Grasping modes	Principle and derived modes	Application scenarios	Experimental demonstration
Closing–opening grasping actions enabled by Arms 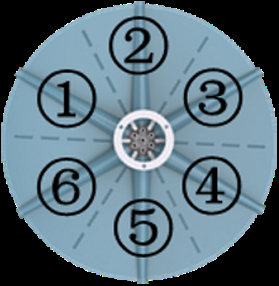	Principle II 1–4 2–5 3–6	Passable objects	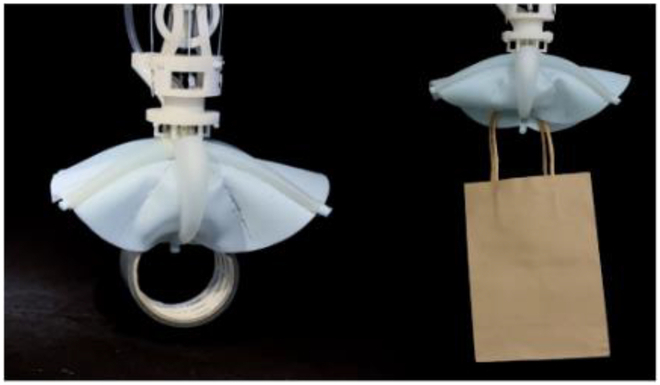
	Principle IV 12–45 23–56 34–61	Elongated objects	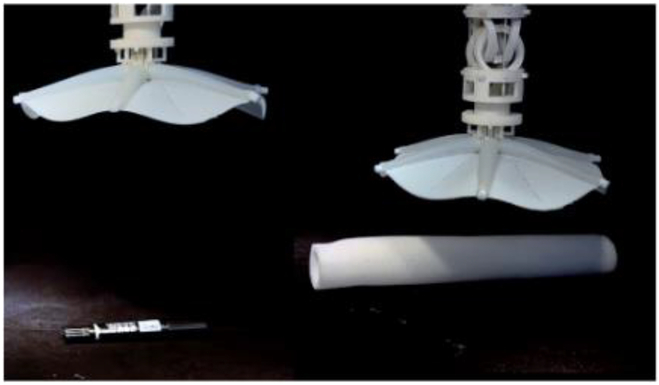
	Principle VI	Small irregular and multiple object	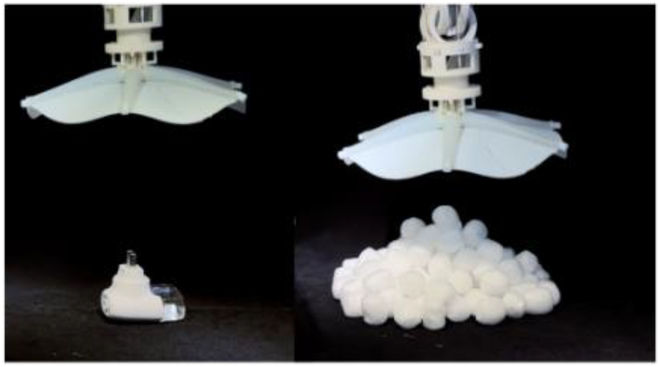
Suctioned grasping action enabled by suckers	Supporting any modes	Planar and curved objects	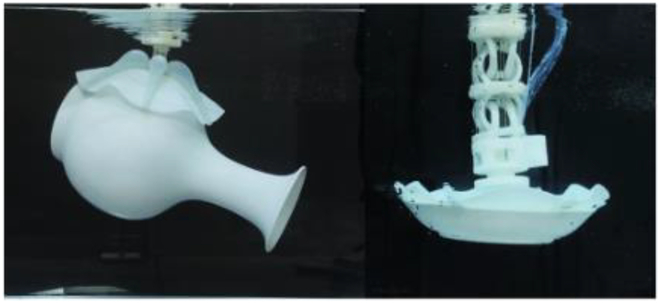

### Multimodal adaptive grasping under the water

The octopus-mimicking soft gripper is combined with a robotic arm for dexterous manipulation of underwater objects. We explored gripper applications in the application scenarios (Table 1 and Movie S5). In principles II, IV, and VI, all Arms are controlled using a pressure of 120 kPa to complete the grasping of the object. In suction grasping mode, the Arm does not actuate and directly performs suction grasping when facing flat objects. The Arm actuates until the suckers can touch the surface of the curved object for suction grasping when grasping large curved objects. Actuation principle II can be applied to grasping objects with handles through which the symmetrical fingers can pass. The actuation principle IV can output a sizeable grasping force to realize the grasping of various elongated objects. The grasping patterns derived from drive principles II and IV enable the gripper to grasp objects at arbitrary angles (Fig. S13 and Movie S6). The 6-Arm bending can realize the grasping of various objects smaller than the diameter of the gripper and the ability to grasp multiple objects (maximum number: 24, diameter: 2 cm) simultaneously. The suction mode can support the actuation principles (namely, II, IV, and VI) and realize the grasping of planar and curved objects. The Adhesion ON-OFF capability of the sucker (Fig. 2E) can effortlessly realize the switch from grasping to releasing.

**Fig. 4.**
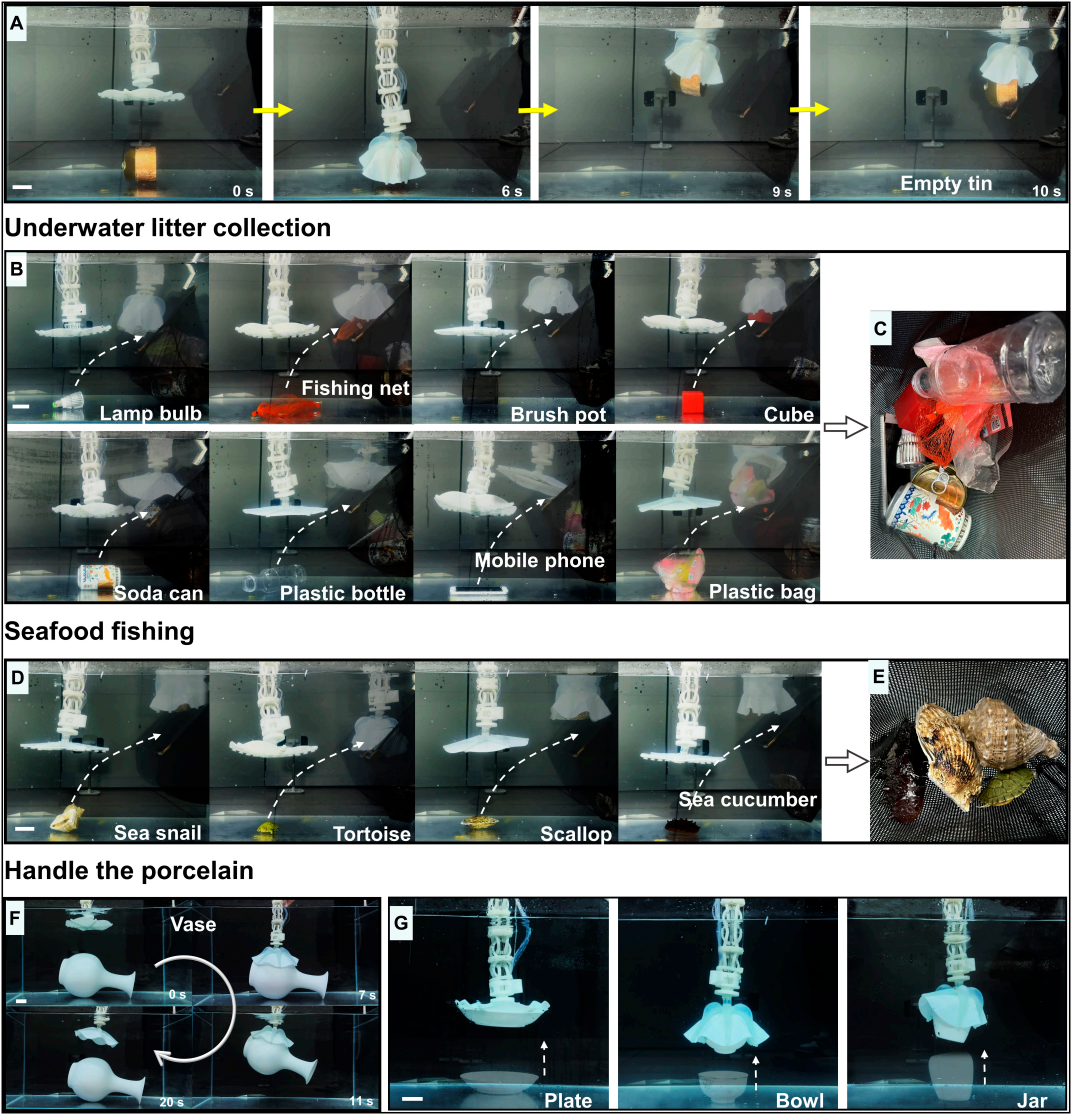
Dexterous manipulation of the soft gripper in underwater scenarios. (A) Illustration of the soft gripper grasping and collecting objects. Soft grippers approach objects–grasp–lift–release them into a storage bag. (B) Several common types of underwater litter collection. (C) Grab a light bulb, fishing net, brush pot, cube, soda can, plastic bottle, mobile phone, and plastic bag, respectively, and put them into the storage bag. (D) Seafood fishing. (E) Grab sea snail, tortoise, scallop, and sea cucumber and put them into the storage bag. (F) The soft gripper is driven by actuation principle VI and the suction mode to gently pick up and put down the vase underwater. (G) Handle the porcelain (plate, bowl, and jar) gently underwater. Scale bar, 10 mm.

We simulated several potential application scenarios underwater: litter collection, seafood fishing, and gently handling porcelain in underwater archeology (Fig. 4 and Movie S6). Figure 4A shows the dexterous manipulation of the grasping process, approaching an object–grasping–lifting–releasing into a storage bag. Figure 4B shows the collection of several common types of underwater litter pollutants. The gripper grabbed and put objects into storage bags one after another, as shown in Fig. 4C, such as a light bulb, fishing net, brush pot, cube, soda can, plastic bottle, mobile phone, and plastic bag. Figure 4D shows the seafood fishing process, grasping sea snails, tortoises, scallops, and sea cucumbers, respectively (Fig. 4E). Figure 4F and G shows the dexterous manipulation of the grasping process on fragile porcelain. The gripper’s soft and flexible characteristics ensure the gentle handling of porcelain and other fragile objects during grasping. The gripper performs the incredible task of grasping porcelain (vase), as shown in Fig. 4F, which is significantly larger than its size. In addition, the plate, bowl, and jar handling are shown in Fig. 4G, respectively.

### Omnidirectional motions of the gripper

**Fig. 5.**
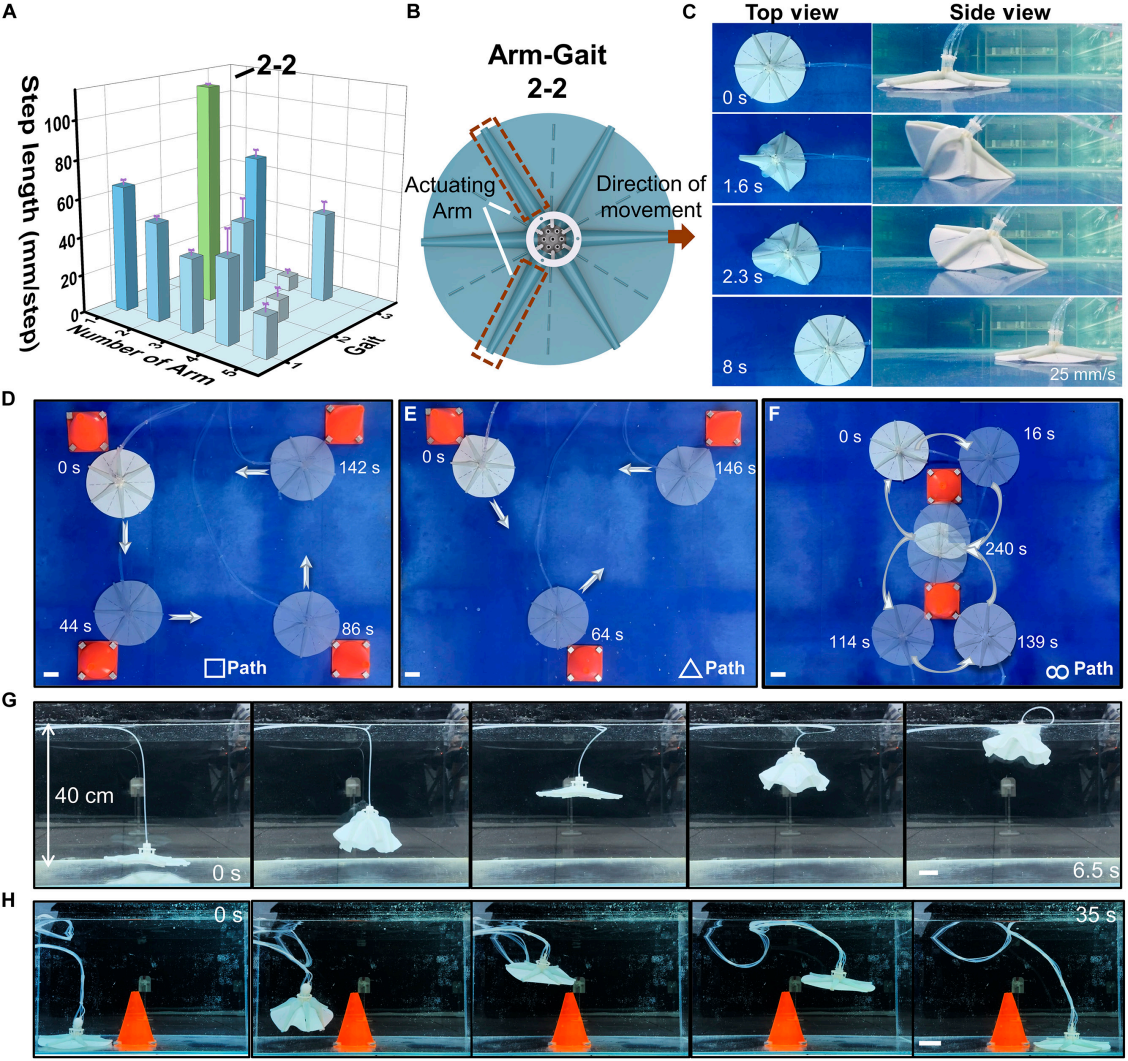
Movement performance of the gripper. (A) Step lengths corresponding to gaits produced by actuating different numbers of Arms. The motion schematics for each gait can be found in Fig. S14. Error bars show standard deviation from 4 tests. (B) Illustration of the fastest gait. This gait is achieved by actuating any 2 Arms separated by an unactuated Arm in between and moving in a given direction. (C) Top and side views of gait 2-2 in progress. The maximum speed at which the gripper crawls is 25 mm/s. (D to F) The path that the gripper performs agile omnidirectional crawling underwater. It can realize the crawling of □ (D), △ (E), and 8 (F) paths, respectively. (G) Vertical swimming is achieved by actuating the 6 Arms of the soft gripper simultaneously. (H) Inclined swimming is achieved by actuating different Arms during vertical swimming to apply perturbations, turning off the pump, and falling in a desired direction. Swimming in 3D enables swimming to the top and across objects while crawling. Scale bar, 10 mm.

To explore the crawling performance of the gripper, we tried all possible combinations of gaits from 1 Arm to 6 Arms. The actuation of one Arm can produce one gait; 2 Arms can produce 3 gaits; 3 Arms can produce 3 gaits; 4 Arms can produce 3 gaits; and 5 Arms can produce one gait. Each gait takes a single full Arm bending as a step-in length. The test results of each gait are shown in Fig. 5A. The gait of the gripper with the fastest crawling speed is actuated by any 2 Arms separated by an unactuated Arm in between, as shown in Fig. 5B. Schematic representations of the remaining gaits are given in Fig. S14. The top and side views of the fastest gait are shown in Fig. 5C. The gripper glides in one direction by applying positive pressure to the Arm to maximum flexion. Then, the pump is turned off to achieve underwater crawling while the Arm recovers. The position of the pipe will affect the movement speed of the gripper (Fig. S15). The maximum speed of the gripper hand when the pipe is located behind is smaller than the maximum speed when the pipe is located in front. The maximum speed at which the gripper crawls is 25 mm/s. The gripper’s symmetrical design enables it to move in 6 directional capacities. Figure 5D to F shows the omnidirectional crawling performance of the gripper with agility (a soft gripper that crawls underwater can drag pipes). The gripper can realize the crawling of “**□**”, “**△**”, and “8” paths, respectively (Movie S7). Besides crawling underwater, the gripper can swim vertically and diagonally (Movie S8). Note that the density of the gripper is adjusted to be close to that of water to minimize the influence of gravity. The gripper can swim vertically like an octopus by simultaneously actuating 6 Arms [42] (Fig. 5G and Movie S8). Different Arms are actuated to exert disturbance during vertical swimming, and inclined swimming can be achieved by free-falling the gripper after turning off the pump. Swimming in 3D enables swimming to the top and across objects while crawling (Fig. 5H and Movie S8). When crawling close to the object, the vertical swimming mode is activated to rise to a position higher than the height of the object, and then the front arm is driven to exert motion interference to make the object fall obliquely and directly above the object. After that, the vertical swimming mode is activated again, and the soft gripper steps over the object and falls freely to complete the task.

### Manipulation task: Releasing and grasping in confined space

**Fig. 6.**
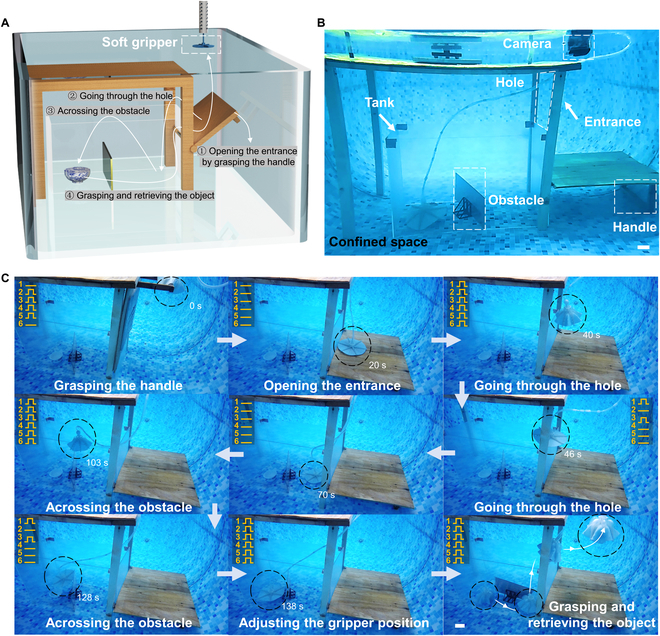
Grasping in confined space. (A and B) Confined space with only one entrance and an object inside that cannot be touched by the robotic arm. (C) Illustration of soft gripper moving and grasping. The circular dashed line represents the position of the soft gripper. First, release the soft gripper and grasp the handle to open the entrance. The soft gripper then goes through the hole and reaches the inside of the tank. Next, across the obstacle inside the tank to reach the object. Finally, adjust the position of the soft gripper to complete the grasping and recycling of the object. Scale bar, 40 mm. The yellow words represent different Arms and the waveforms of the control signal.

We demonstrated the possibility of using the gripper in confined underwater space because of the gripper’s diverse grasping modes and excellent movement performance. The soft gripper can even perform tasks that are out of reach of the robotic arm by expanding the application space (Fig. S16). As shown in Fig. 6A and B, we put the gripper to a challenge to perform grasping in a complex environment. So, we constructed a complex underwater confined space in a 1.5-m deep pool. Figure 6A shows the step-by-step procedure of the task, and Fig. 6B shows the perspectives view of the experimental setup. The robotic arm cannot enter the tank to grasp directly because there is only one entrance to this confined space, which can be opened via a handle. The walls of the tank are defined as fixed obstacles, and there are moveable obstacles placed inside the tank to complicate the environment further. The gripper must enter through the hole and then swim over the movable obstacle to reach the object inside the tank. As shown in Fig. 6C, the object can be approached and grasped by controlling the movement of the soft gripper (Movie S9). The steps of this intricate grasping task are as follows. First, the gripper is unmounted from the robotic arm, the handle is grasped, and the handle is pulled on to open the entrance. Second, the vertical swimming mode is turned on to the appropriate height and the inclined swimming mode is driven across the hole. Third, the inclined swimming mode is turned on to cross the obstacle inside the tank. Fourth, the gripper turns on crawling mode to approach the object and swim above the object. Finally, the position of the gripper is adjusted above the object, the object is grasped, and then the pipeline is retrieved to complete the grasping of the object. During the retrieval process, neither movable nor fixed obstacles affect the stability of the grasp. In future work, a panoramic or small tethered camera can be added to the gripper to facilitate exploration and grasping tasks that are out of the global camera’s vision. This feature will significantly increase the operational space and application scenarios for robots.

## Discussion

We have introduced an octopus-inspired soft gripper that integrates adaptive grasping, omnidirectional crawling, and 3D swimming to tackle the challenges of exploration and adaptive grasping in underwater unstructured environments. The soft gripper enables the control of individual Arms. When the soft gripper is mounted on the robotic arm, it offers 4 actuation principles: II, IV, VI, and suction mode. The modes of actuating arbitrary symmetrical 2 and 4 Arms derived from actuation principles II and IV allow grasping the target object at any placement angle without twisting the gripper. The contribution of this soft gripper is unusual compared to existing research on forked, symmetric grippers [23,43], which we expect to lead to significantly improved grasping efficiency. This gripper achieves excellent grasping performance for the extensive range of adaptive grasping because of features such as the ability of the Arm to generate joints at any position and the combination of 4 actuation principles. The grasping performance has been verified in the experiment, and it can realize the grasping of irregular small objects, long strip objects, objects larger than the diameter of the gripper, multiple small objects, and flat, curved, rigid, and flexible objects. Therefore, the soft gripper can be essential in labor-intensive tasks, such as grasping various forms of underwater litter, seafood, and porcelains. Especially for the variety of forms of underwater litter generated by humans, the multimodal grasping mode can be fully utilized. As shown in Fig. 4B, we have continuously grasped 8 different types of common underwater litter in one go, which is beneficial for significantly improving the efficiency of underwater litter collection. The flexibility of the gripper material allows for gentle gripping, which is very friendly to seafood and fragile objects. We compared several representative grippers, as shown in Table 2. Our soft gripper achieves a greater variety of grasping modes to handle diverse types of objects. In the future work, the gripper’s grasping will be improved by embedding variable stiffness material on the Arm, and the gripper can be transformed from soft to stiff according to the task. Recent research has demonstrated the possibility of tunable stiffness of materials [44,45]. This feature can allow the pump system to be powered OFF after activation (no power is consumed during grasping/manipulation), avoiding the pump system being ON all the time to save energy and improve grip stability.

**Table 2.** Comparison between the proposed soft gripper and the other grippers. Larger than the gripper size: ① Objects with handles: Such as cups, paper bags, and tape. ② Long, cylindrical objects: Similar to rods, markers, and plastic bottles. ③ Objects that can be placed at any angle. ④ Large flat or curved surfaces: Like vases or plates. Smaller than the gripper size: ⑤ Small, irregular objects: Such as light bulbs or seashells. ⑥ Simultaneously grasping multiple small objects.

Types Performance	This work	Finger-type gripper [24,55]	Suction cup-type gripper [12,56]	Wrap-around gripper [25,31,57]	Jamming effect gripper [32,33]	Finger with suction cup hybrid gripper [58,59]
Multi-modal grasping	√					**√**
Number of grasping modes	8	1	1	1	1	3
Types of grasped objects	①②③④⑤⑥	①②⑤	④	⑤⑥	①⑤	①②⑤
Controllable suction	√					
Omnidirectional crawling	√					
3D maneuvering	√					
Detaching from robotic arm work	√					

The suckers distributed on the Arm can realize the pre-adhesion and reversible adhesion function when picking up and releasing various underwater objects, which is a crucial combination for realizing underwater dexterous manipulation. Through the combination of flexible grasping by the Arm and reversible adhesion of the sucker, it can nondestructively grasp the vase, which is much larger than the size of the gripper, and handle it gently, which is very beneficial for the grasping of porcelain in underwater archaeology. It is worth noting that each sucker can form independent compartments and achieve redundant adhesion just like its biological counterpart to improve the suction mode’s robustness. The pre-adhesion of the suckers is expected to improve the success rate of suction grasping, and there are future opportunities to integrate haptic feedback [46] into this system, which will allow adjustment of the control scheme for customizable underwater manipulation. In addition, pressure sensors can be added to the sucker to sense and describe the object’s contour, which is an excellent direction for working in turbid underwater environments.

Further, our soft gripper can perform grasping and retrieving in unstructured underwater environments. The soft gripper can be switched between attached and detached from the robotic arm, and the ability of the gripper to move away from the robotic arm for controlled movement and grasping is exciting. Benefiting from independently controlled Arms and the symmetry of the gripper design, the gripper can crawl and swim in 3D along the directions of the 6 Arms, respectively. Omnidirectional movement allows the soft gripper to move in narrow spaces, enabling it to function in confined spaces where robots or robotic arms cannot reach. This feature has vital application value for exploring unstructured environments such as shipwrecks or some gaps/cracks/vents underwater. As shown in Table 2, we are the first to introduce a gripper that can operate independently of the robot arm by increasing its range. In future work, researching the operational capabilities of underwater robots carrying multiple grippers, as well as the interaction of swarms of grippers, presents an exciting scientific problem. This allows for the study of individual interactions and their impact on swarm performance and overall function. To implement such controlled team operations, existing technologies, including acoustic waves [47], wireless optical communication [48], and electric field communication [49], can be used for communication. This research will facilitate the development of autonomous robots for exploration operations in complex, unstructured underwater environments.

## Materials and Methods

### Fabrication and assembly of the soft gripper

The detailed fabrication process is shown in Figs. S6 and S7. The first step of the fabrication was the design and fabrication of the mold. The modular mold was designed in SolidWorks. The computer-aided design (CAD) models were sliced with Materialise Magics Software and printed by Stereo Lithography Apparatus (SLA) 3D printer. The printing material was photosensitive resin. The printer (SLA550) and printing materials were purchased from Zrapid Tech Co. Ltd. The multi-step molding and casting process was used to fabricate the suckers and Arms. The mold used to fabricate the sucker was divided into 4 parts ①②_R_②_L_③, mold ③ was used to form the inner space of the sucker, and mold ①②_R_②_L_ was used to determine the outer contour of the sucker. First, ②_R_②_L_ and ③ were assembled, and then silicone elastomer (Dragon Skin 20, Smooth-On Inc., PA) was poured into the mold and degassed in a vacuum chamber for 10 min. Mold ① was then covered, and the elastomer was left at room temperature for 6 h to cure (Fig. S6A to D). The mold fabricating the tapered actuator was divided into 4 parts: ④⑤⑥⑦. The channel formed by mold ⑤ was used to connect the sucker to form suction. Mold ⑥ was used to form the inner chamber of the Arm. First, ⑤⑥⑦ were assembled, the elastomer was poured into the mold, and it was degassed in a vacuum chamber for 10 min. Mold ④ was then covered, and the elastomer was cured at room temperature for 6 h (Fig. S6E to H). The mold (Fig. S7A) was used to cast the ventral membrane, and the fabricating process was the same as that of the tapered actuator. After the elastomer was cured, the mold was removed. Next, the fabricated sucker and Arm were sealed with an adhesive (Sil-Poxy, Smooth-On Inc., PA) to form a complete Arm (Fig. S7B). Similarly, the 6 Arms and the ventral membrane are also sealed with the same process to form a complete gripper (Fig. S7C), and the fabricated soft gripper is shown in Fig. S7D.

### Design and fabrication of gripper accessories

The accessories of the gripper, shown in Fig. S8, are the robotic arm, connector, and clamp. They were all designed in SolidWorks and printed with an SLA550 stereolithography printer. The continuum robotic arm consists of serially connected segments acting as compliant Cardan joints. Each segment contains 2 sets of flexures enabling bending around the *Z* axis and *Y* axis of the joint. The continuum robotic arm is actuated by 4 cables passing through the 4 passage holes in the guidance disk, which makes this robot adaptable and flexible. This design suits applications requiring intricate motion and navigation in constrained spaces. The top of the connector is attached to the robotic arm’s end effector, while the gripper is mounted on the bottom side of the connector. It is designed with a hollow structure in the middle to allow the pipe to pass through and out from one side. The design of the clamp system used to control the gripper is shown in the figure. From the vertical view, we can see that there are 6 Arm control pipes, and each pipe controls one Arm to achieve independent control. As shown in the sectional view (Fig. S8), the pipe for controlling the sucker is placed under the pipe for controlling the Arm, and the central pipe realizes the unified control of positive- and negative-pressure switching of the sucker. Electromagnets are employed to assemble the clamp and connector of the gripper to realize switching between attached and detached from the robotic arm.

### Grasping force measurement and adhesion tests

Test of the bending force of a single Arm: As shown in Fig. 2D, the end of the dynamometer is tested against each sucker on the Arm. The actuation pressure was set at 140 kPa, while the output in finger bending force was recorded (Fig. S10).

1. Test of the grip strength of the gripper: The grip strength of the grippers corresponding to actuation principles II, IV, and VI were tested. First, the gripper is fixed on the clamp, as shown in Fig. S10A, and then the gripper is driven to wrap the object with a pressure of 140 kPa placed on the center of it. After that, a linear actuator pulls the grasped object upward at 12.5mm/s until it gets released from the gripper. The dynamometer is attached to the linear actuator on one end and object on the other. It records the real-time output of the force required for the gripper to keep the object in grip from the initial state to the half-detached state until entirely detached from the gripper. The objects involved in the grip strength test were cylinders with diameters of 90, 120, and 200 mm and a length of 200 mm (Fig. S10B and C) and spheres with diameters of 90, 120, and 200 mm (Fig. S10D). The evaluation of the output of the 3 actuation principles facing different objects is shown in Fig. S11. The grasping force output will increase if more Arms are actuated for objects with smaller diameters.

2. Suction test in suction mode: The suction test experiment with the gripper was performed underwater (Fig. S12A and Movie S5). The experiments of the gripper detaching from the substrate from the center and the edge were tested, respectively, and the real-time output graph is shown in Fig. S11B. The substrate was covered on the gripper, the negative pressure −60 kPa was started and maintained at the same time, then the substrate was lifted at a speed of 12.5 mm/s until the sucker is entirely detached from the substrate, and the real-time output was recorded in the suction force throughout the process.

### Finite element method simulations

A static nonlinear finite element simulation was performed using the finite element software Abaqus/Standard (Simula, Providence, RI) to predict the Arm’s bending angle and the tip’s motion trajectory to validate the experimental results with the gripper. The model was constructed using a 4-node linear tetrahedral element (Abaqus element type C3D4H), and the material response was captured using the Yeon hyperplastic material model [50]. All material parameters are measured by mechanical property tests (Mark-10 F105; Fig. S17), and the Dragon skin material coefficients are C10 = 0.06066220764, C20 = 0.00223876341, and C30 = 1.853197581E00. The simulation results agreed with the experimental results, and numerical simulation can be carried out to explore the parameter design space comprehensively and quickly. In addition, the positive pressure and negative pressure of the sucker were also predicted to predict whether the protrusion can be generated when the positive pressure is driven, which provides a basis for the design of the sucker.

### Control system design

The 6 Arms and suckers are driven by 7 geared pumps (GGMN 1200, Korea, the actuating medium is water), and dual H-Bridge (L298N) modules are used to control positive- and negative-pressure switching control 6 Arms and suckers (Fig. S18). Arduino sends the signal to L298N to control each pump. A regulated source powers both Arduino and L298N through a voltage regulation module. The suckers have 2 modes of actuation: In the first mode, a suction grasping action enabled by suckers, negative pressure activates suckers to grasp flat objects. After the grasp is completed, positive pressure activates the suckers to release the object. In the second mode, a closing–opening grasping action enabled by Arms, suckers are controlled together with the Arms. When the Arms are activated for grasping, negative pressure activates suckers to assist in grasping objects. A positive pressure activates the suckers to release the object when the Arm returns to its initial state after the grasping is completed. The overall weight of the control system is 1 kg, which is convenient to carry on a small underwater robot.

## Data Availability

All data needed to evaluate the conclusions in the paper are present in the paper and/or the Supplementary Materials.

## Supplementary Materials

Supplementary 1 Figs. S1 to S18 Movies S1 to S9 References [51–54]
