# Supplementary material for: Octopus-Inspired Underwater Soft Robotic Gripper with Crawling and Swimming Capabilities
Source: Research (Wash D C). 2024 Aug 28;7:0456. doi: 10.34133/research.0456 (PMC11350063; doi:10.34133/research.0456)
Supplement: Supplementary 1 — Figs. S1 to S18 Movies S1 to S9 References [51–54] [file research.0456.f1.zip › Supplementary Materials.docx]

Supplementary Materials

**Octopus-inspired underwater soft robotic gripper with crawling and swimming capabilities**

Mingxin Wu^1^, Waqar Hussain Afridi^1^, Jiaxi Wu^1^, Rahdar Hussain Afridi^1^, Kaiwei Wang^1^, Xingwen Zheng^1^, Chen Wang^1, 2^, Guangming Xie^1, 3^*

^1^State Key Laboratory for Turbulence and Complex Systems, Intelligent Biomimetic Design Lab, College of Engineering, Peking University, Beijing, 100871, China

^2^National Engineering Research Center of Software Engineering, Peking University, Beijing, 100871, China

^3^Institute of Ocean Research, Peking University, Beijing 100871, China

* Corresponding author: Guangming Xie (xiegming@pku.edu.cn)

This file includes:

Supplementary Text

Fig. S1. Analytical model for the design of the Arm

Fig. S2. Adaptive bending capability of the Arm.

Fig. S3. Two working states of the suckers.

Fig. S4. Adhesion of individual Arms.

Fig. S5. Comparison of the force of breaking the suckers and the arm and the suction force of the suckers.

Fig. S6. Fabrication of the sucker and Arm.

Fig. S7. Fabrication process of the soft gripper.

Fig. S8. The accessories of the gripper include the continuum robotic arm, connector and clamp.

Fig. S9. Comparison of the behavior of soft grippers with and without rectangular gaps.

Fig. S10. The experimental setup and real-time output for the grasping force test.

Fig. S11. The evaluation of the output of the three actuation principles facing different objects.

Fig. S112. Suction test in suction mode.

Fig. S13. Adaptive grasping of objects placed at different angles.

Fig. S14. Schematic showing the gait corresponding to Fig. 5B.

Fig. S15. Effect of pipe position on gripper movement speed within the same time period.

Fig. S16. Demonstration of expanding application space without the limitations of robotic arm.

Fig. S17. Material behavior.

Fig. S18. Block diagram of the control interface in the soft gripper.

Movie S1. Continuous bending ability of the Arm.

Movie S2. The actual operation diagram of the sucker.

Movie S3. Single Arm peeled vertically from different positions.

Movie S4. The gripper peeled vertically from different positions.

Movie S5. The application of gripper in the different scenarios.

Movie S6. Dexterous manipulation of the soft gripper in underwater scenarios.

Movie S7. “**□**”, “**△**”and “8” underwater crawling paths.

Movie S8. 3D swimming of the soft gripper.

Movie S9. Releasing, moving, and grasping underwater.


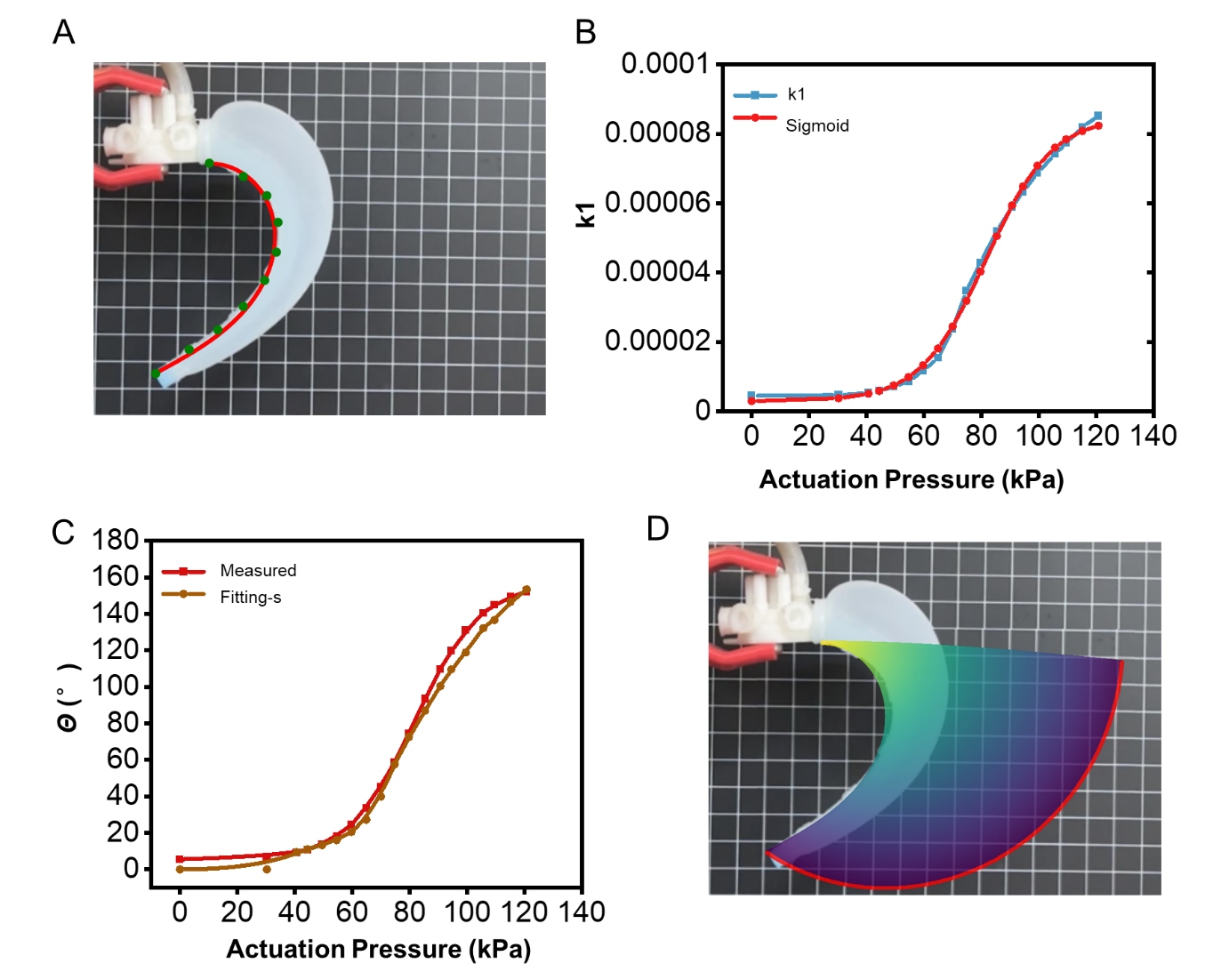


**Fig. S1.** Analytical model for the design of the Arm.

Current soft continuous robots mostly are cylinders or cuboids, and modeled under constant curvature assumption [51][52]. However, the single finger utilized in our work is conical structure, whose bending is not constant curvature, thus the theoretical models in [1][2] is not suitable for our work. Refer to [53][54], Euler curves are adapted in our work to establish the variable curvature model. The curvature $k(s)$ of a Euler curve increases linearly with the arc length $s$,

$k(s)=k_{0}+k_{1}s$ (1)

Where, $k_{0}$ is the initial curvature ($k_{0}=0$ in our case), $k_{1}$ is the rate of curvature increase.

$x(s)=\int_{0}^{s} cos(\theta(s))ds$ (2)

$y(s)=\int_{0}^{s} sin(\theta(s))ds$ (3)

$\theta(s)=\int_{0}^{s} k(s)ds$ (4)

However, the above three equations involve integrals, which are difficult for computers to solve, so we solve them numerically with the help of differential equations. For the Euler curve whose initial position and angle are $(x_{0},y_{0},\theta_{0})$, the curvature change function satisfies equation (1), the positions and orientation of each point on it satisfy

$\dot{x}(s)=cos(\theta(s))$ (5)

$\dot{y}(s)=sin(\theta(s))$ (6)

$\dot{\theta}(s)=k(s)$ (7)

In our case, the starting position$(x_{0},y_{0})$ are the end of the finger, the starting orientation satisfies

$\theta_{0}=\pi-\theta(L)$ (8)

where, $\theta(L)$ is the orientation of the end point ($s=L$) of the Euler curve calculated by equation (4). In the actual calculation, we can first assume $\theta_{0}=0$, and calculate the Euler curve according to equation (5)-(7). After obtaining $\theta(L)$, we rotate the curve with angle of $\pi-\theta(L)$ to obtain the final Euler curve.

In order to evaluate the difference between the Euler curve and the realistic bending curve, we select 10 evenly distributed points on the practical bending picture of the single finger (Fig. S1a), and adapt root-mean-square error (RMSE) to evaluate the fitting error of the Euler curve,

$RMSE=\sqrt{\frac{\sum_{i=1}^{n} d_{i}^{2}}{n}}$ (9)

where, $d_{i}$ is the distance between the selected point $i$ and the closest point to it on the Euler curve.

According to equation (8), we can get the Euler curve parameters $k_{1}$ with smallest RMSE under different pressures, and the relationship between pressure $P$ and $k_{1}$ is shown in Fig. S1b. Then we use the saturation function Sigmoid to fit the $k_{1}-P$ mapping.

The standard Sigmoid function is

$s(x)=\frac{1}{1+e^{-x}}$ (10)

We translate and scale it to better fit the $k_{1}-P$ curve, i.e.,

$s(P)=\frac{c_{2}}{1+e^{-c_{1}(P+c_{3})}}+c_{4}$ (11)

Where, $c_{1}$ and $c_{2}$ are the scaling coefficient, $c_{3}$ and $c_{4}$ are translating coefficient。The fitting results are shown in Fig. S1c, which can basically describe the $k_{1}-P$ mapping well.

Furthermore, according to equation (4), we can obtain the bending angles $\theta=\theta(L)$ under different pressures. Meanwhile, according to the equation (2) and ( 3), we can calculate the motion trajectory of each point on the single finger, and draw the workspace of the finger, as shown in Figure S1d.

**
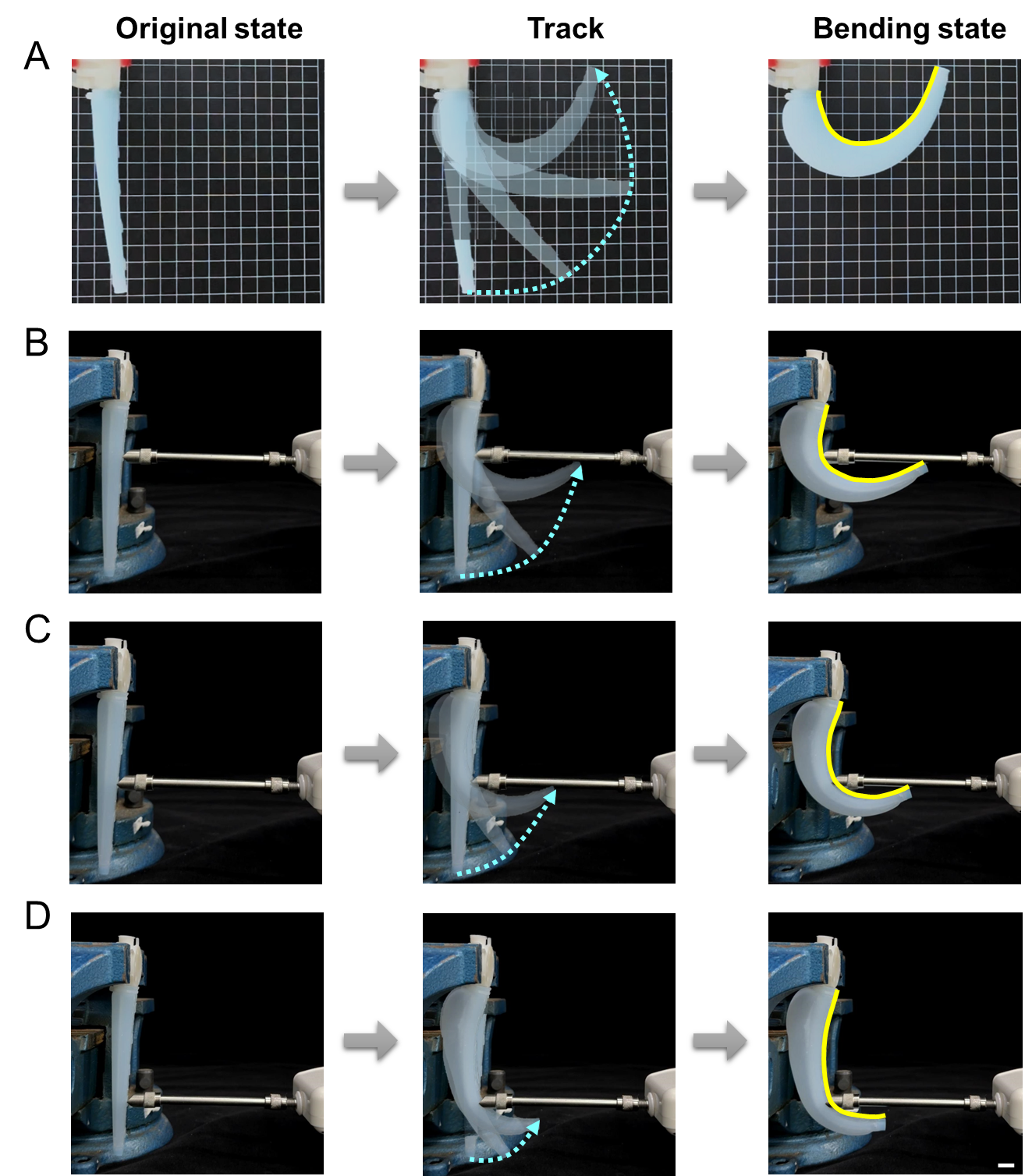
**

**Fig. S2. Adaptive bending capability of the Arm. (A)** The bent state of the Arm without interference. **(B-D)** Different points of the Arm can be disturbed to form different bending states under 140 kPa pressure actuation. This lays the foundation for adaptive grasping with soft grippers. Scale bar, 10 mm.


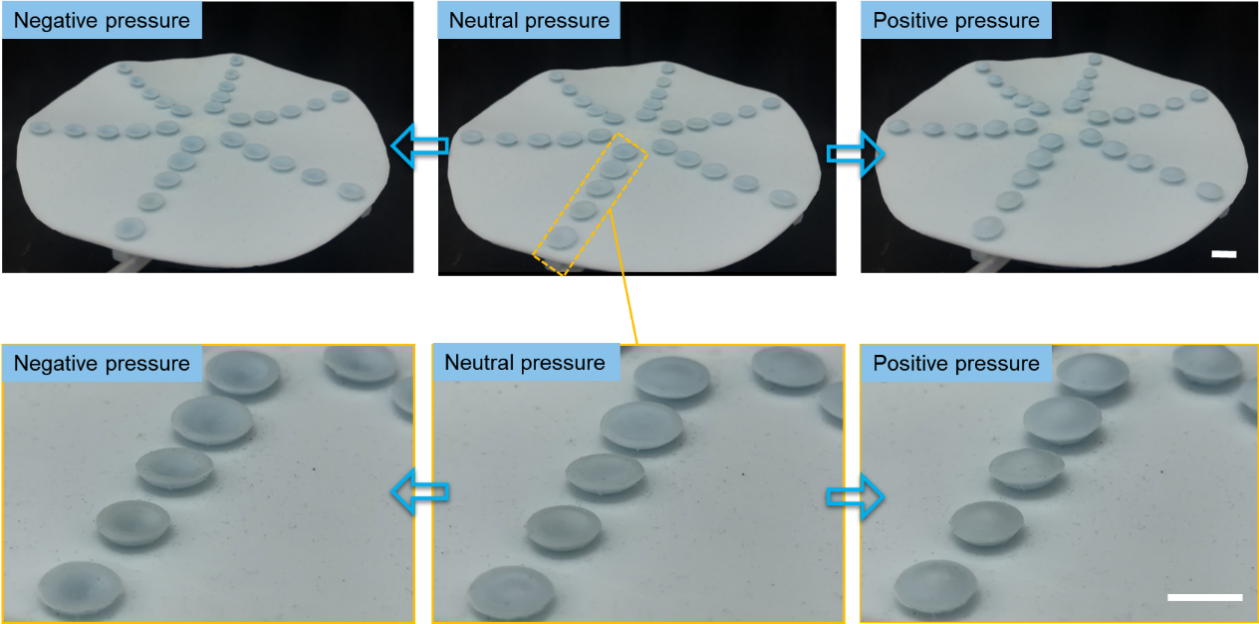


**Fig. S3.** **Two working states of the suckers.** The suckers can produce pre-adhesion under natural pressure, switch the negative pressure to enhance the adsorption force and switch the positive pressure to release the object quickly. Scale bar, 8 mm.


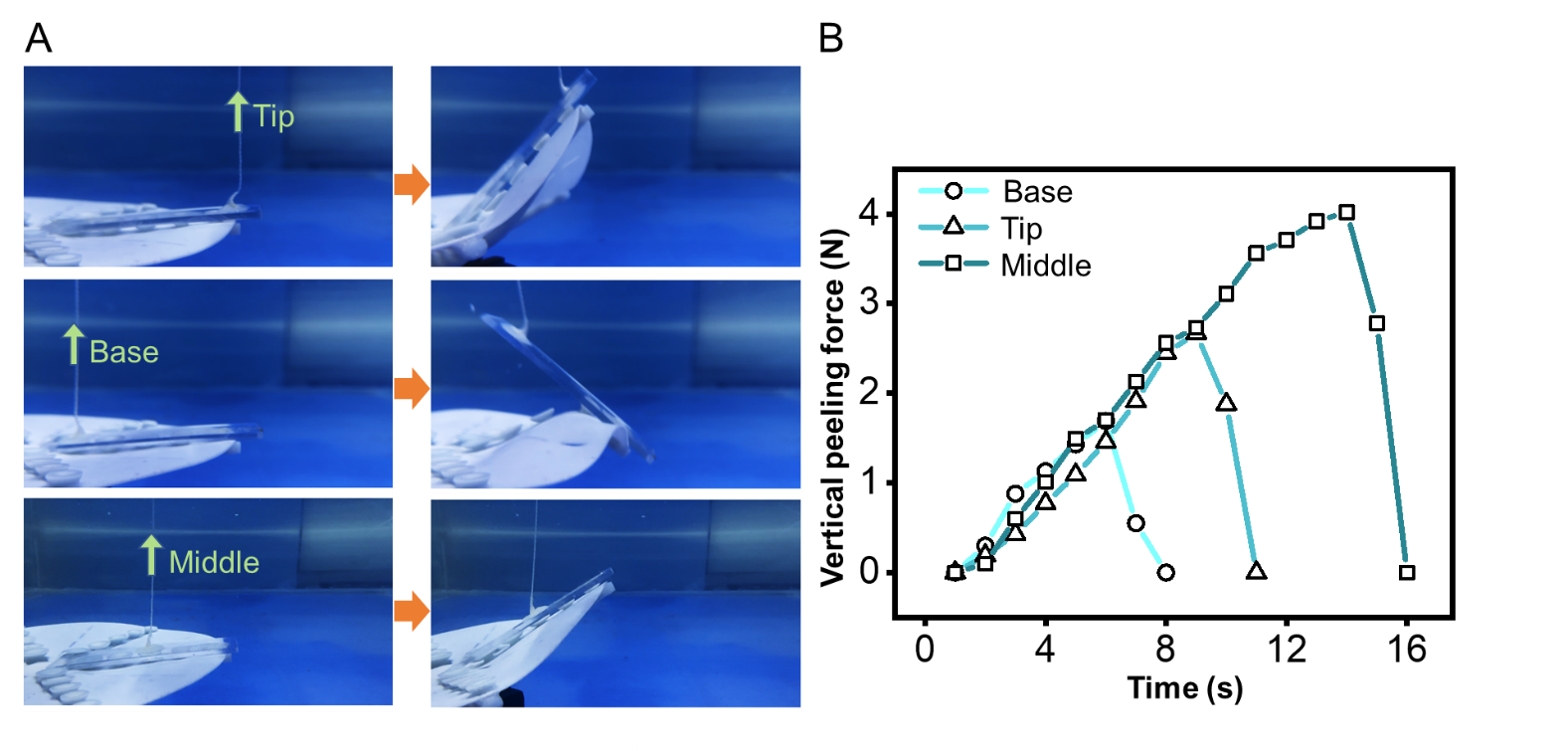


**Fig. S4. Adhesion of individual Arms. (A)** Vertically peel off the plane substrate adhered by the Arm from the tip, middle, and base, respectively. **(B)** The three peeling methods correspond to the adhesion change process of the Arm. Scale bar, 8 mm.

**
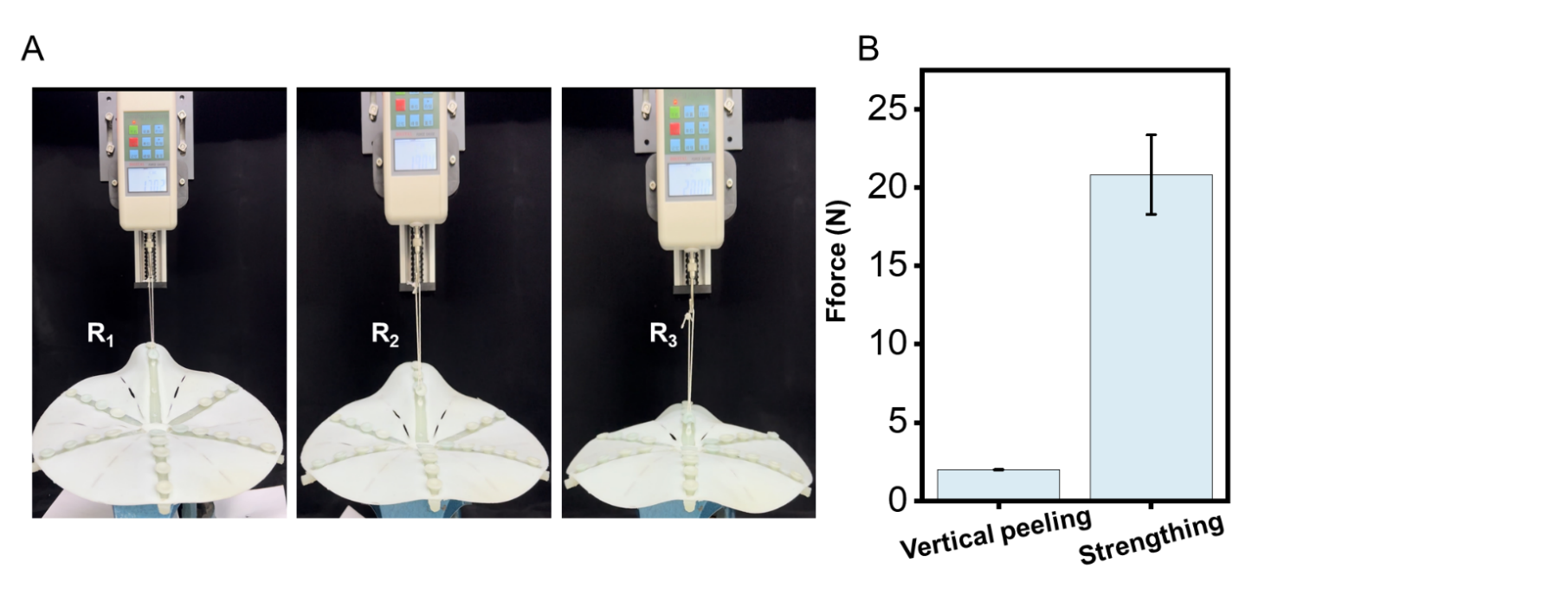
**

**Fig. S5. Comparison of the force of breaking the suckers and the arm and the suction force of the suckers.** (**A**) Schematic diagram of the three-diameter suckers breaking operation. (**B**) Comparison of breaking force and sucker suction force. Error bars show standard deviation from 4 tests.


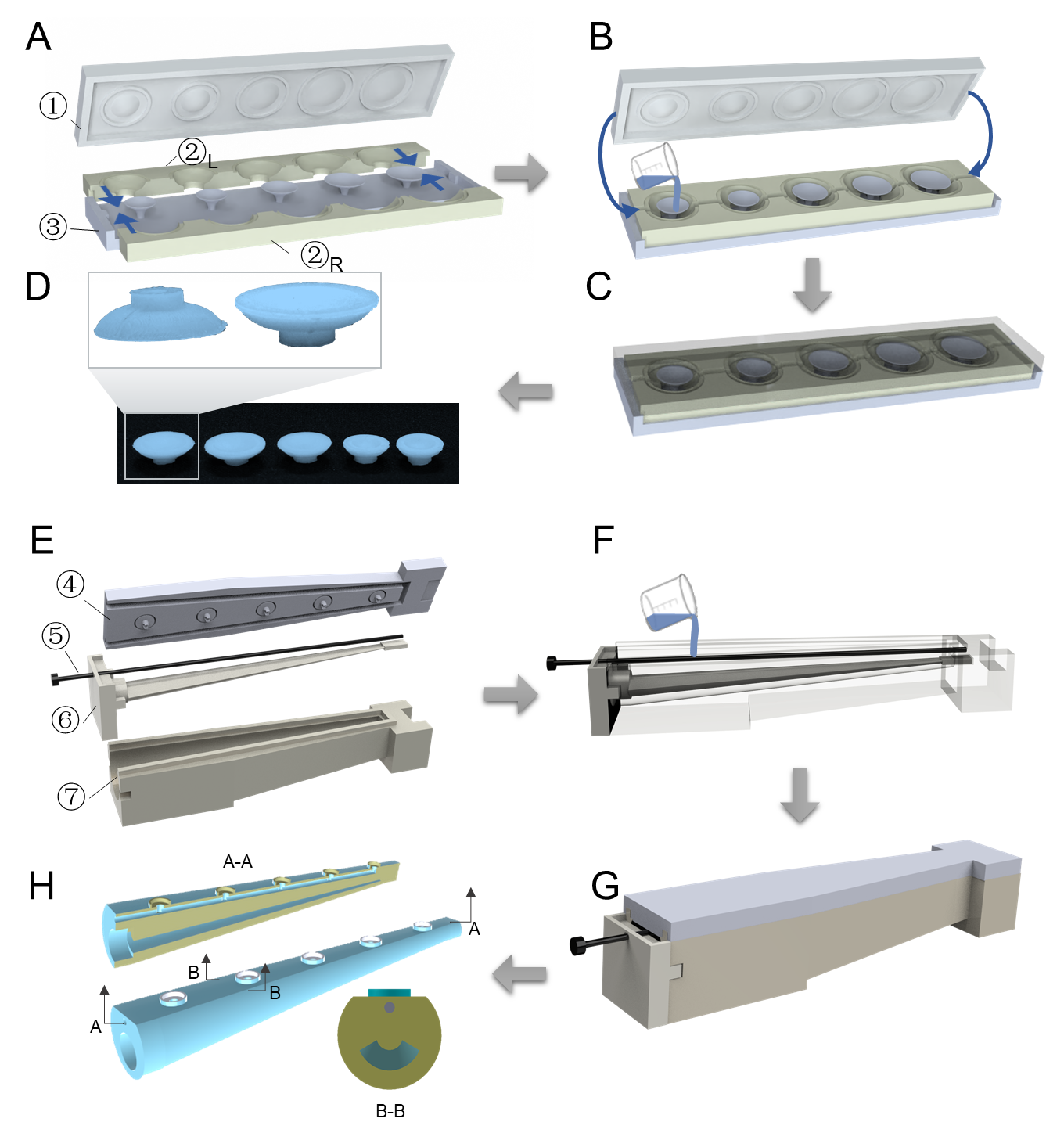


**Fig. S6. Fabrication of the sucker and Arm. (A)** 3D-printed molds are used to cast the sucker. **(B)** Elastomer Dragon Skin 20 is poured into the sucker mold. **(C)** The 3D-printed caps were then laid on the mold to create the shape of the suckers as the elastomer cured. **(D)** The fully cured suckers were complete. **(E)** 3D-printed molds are used to cast the Arm. **(F)** Elastomer Dragon Skin 20 is poured into the Arm mold. **(G)** The 3D-printed caps were then laid on the mold to create the shape of the Arm as the elastomer cured. **(H)** The fully cured Arm was complete. The cross-sectional view shows the arrangement of the inner arch-shaped chamber.


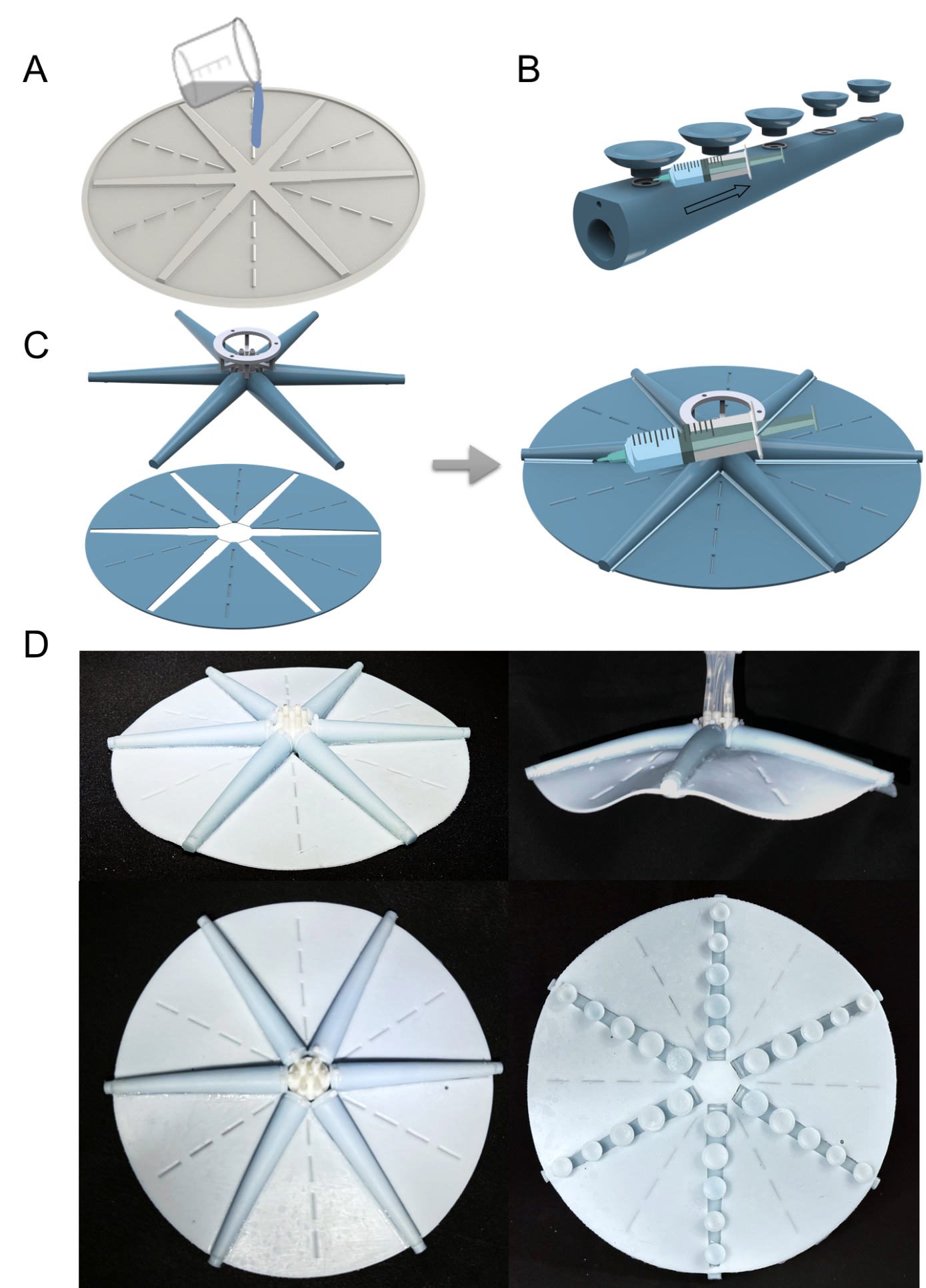


**Fig. S7. Fabrication process of the soft gripper. (A)** 3D-printed molds are used to cast the ventral membrane. **(B)** The fabricated sucker and Arm are sealed with an adhesive (Sil-Poxy, Smooth-On Inc., PA) to form a complete Arm. **(C)** The six Arms and the ventral membrane are sealed to form a complete soft gripper. (**D**) Biomimetic prototype photographs of the soft gripper.


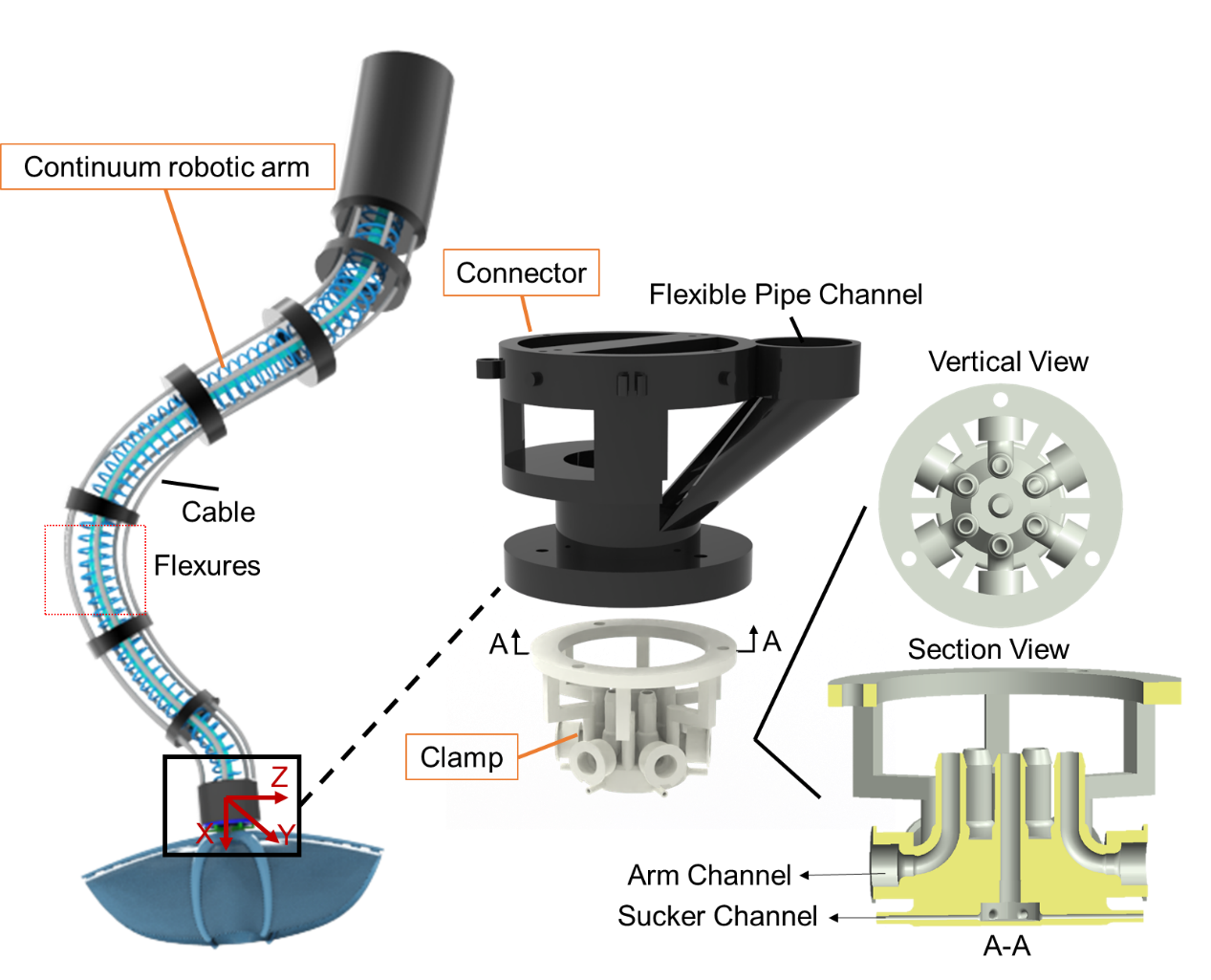


**Fig. S8. The accessories of the gripper include the continuum robotic arm, connector, and clamp.** The continuum robotic arm is used to move the gripper, the connector is used to fix the clamp of the gripper, and the clamp system is used to channel the pressure to the Arms and suckers.

**
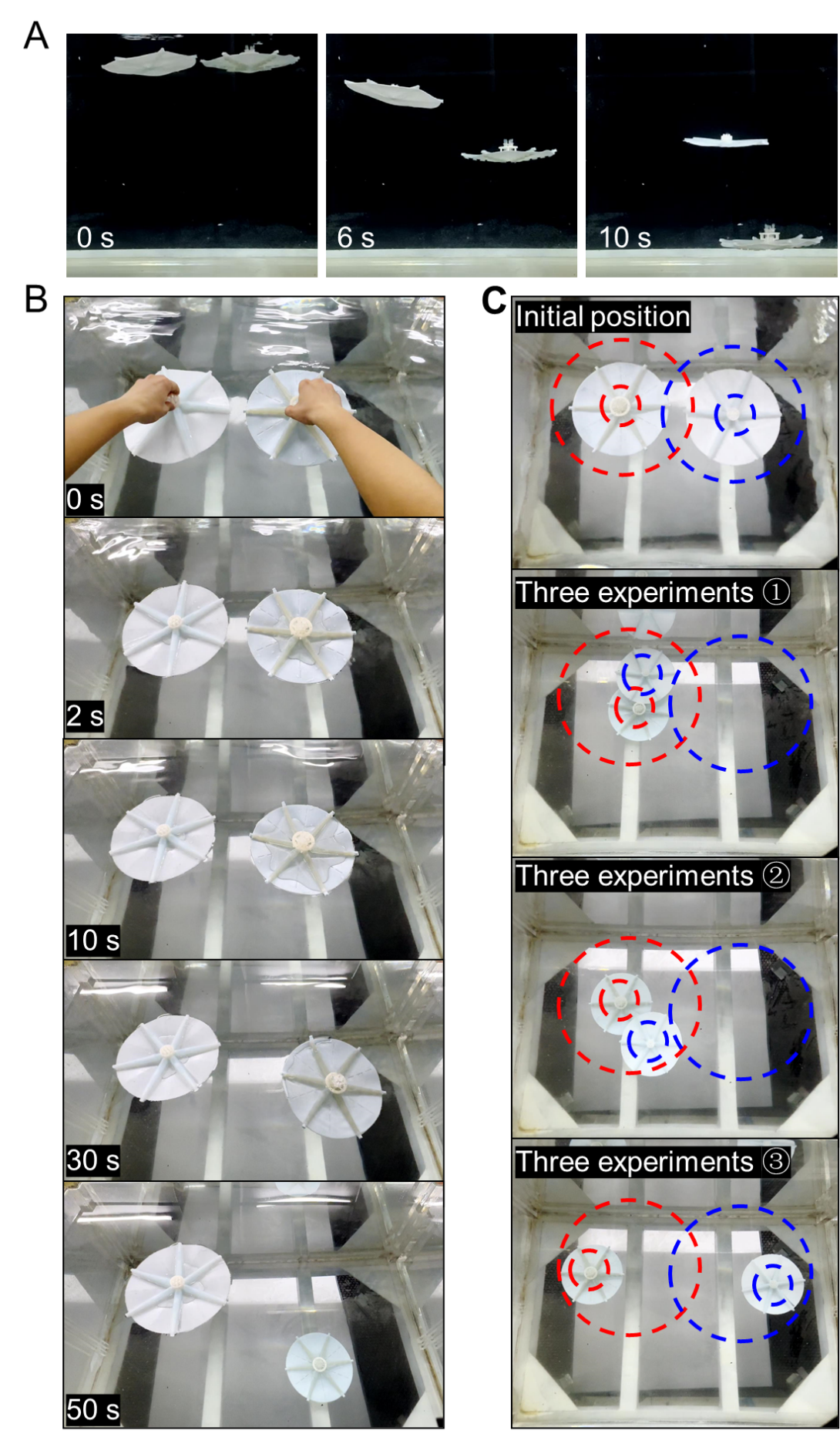
**

**Fig. S9. Comparison of the behavior of soft grippers with and without rectangular gaps.** (**A**) Comparison of the free fall speed of the two grippers when released at the same height underwater. (**B**) Two grippers are placed on the water surface at the same time, and the position of the gripper changes with time. (**C**) Comparison of the landing points of the two grippers at the bottom of the water when they are released simultaneously from the same height in the water.

As shown in Figure S9, by comparing with the gripper without gaps, it is found that the design with rectangular gaps has three advantages: First, the grasper with a rectangular gap has a faster underwater free fall speed (Figure S9a). By releasing both grippers from the same position, the gripper with the rectangular gap reaches the bottom faster. Second, the rectangular gap facilitates gripper release on the water surface. As shown in Figure S9b, the two grippers are placed on the water surface. The gripper with the gap reaches the bottom faster, while the gripper without the rectangular gap floats on the surface. Third, grippers with rectangular gaps have a more stable landing point. Multiple comparison experiments show that the landing point of the gripper with a gap is always within the red circular range. The gripper without a rectangular gap has an unstable landing point, which is not conducive to the fixed-point placement of the gripper. In summary, four rectangular gaps are added to each ventral membrane to have a faster-falling speed and a more stable landing point when the grasping hand is released at a fixed point underwater.


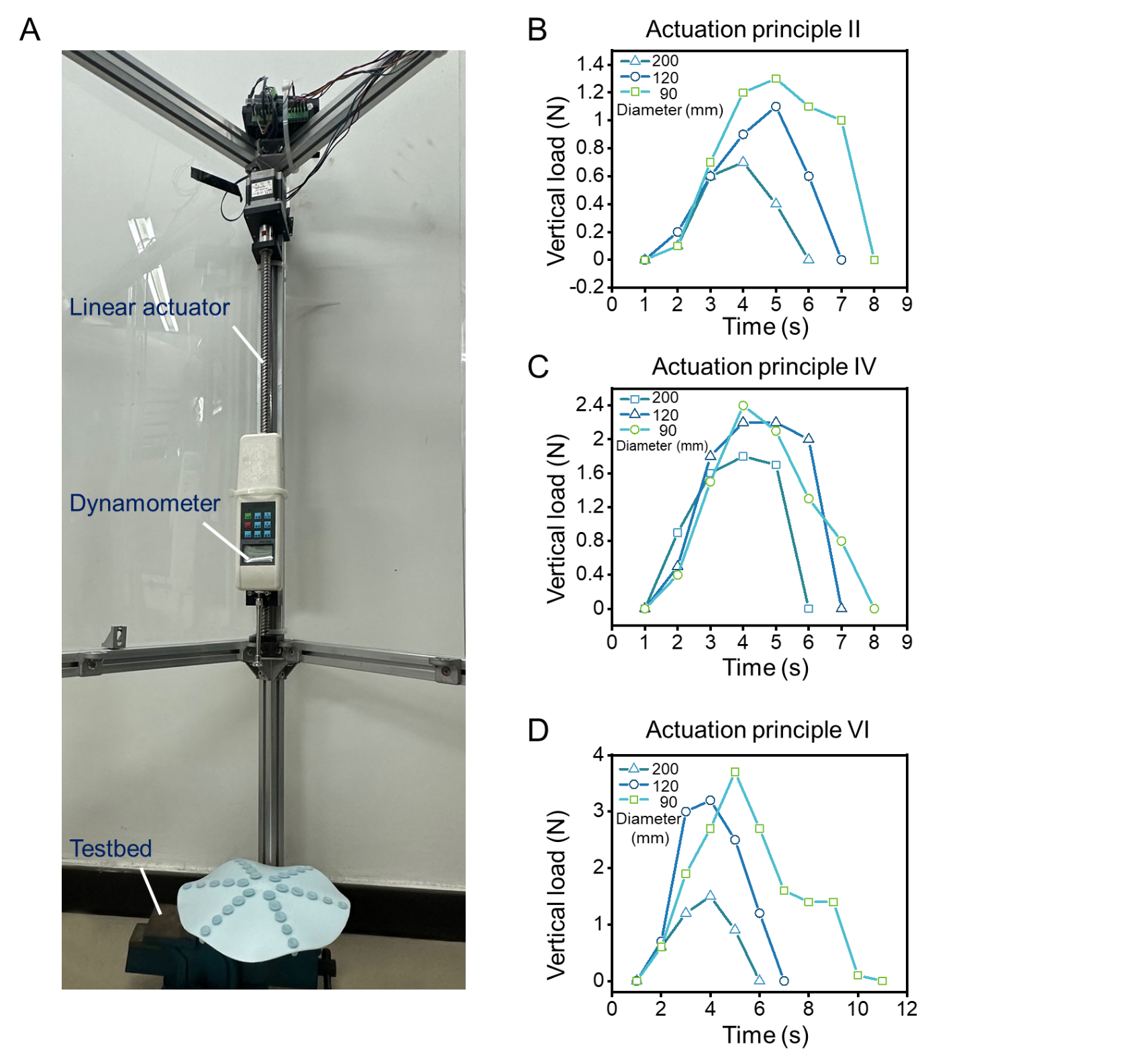


**Fig. S10.** **The experimental setup and real-time output for the grasping force test. (A)** Experimental setup. During these tests, the soft gripper was fixed to the testbed, and the horizontal cylinder was positioned at the center of the gripper. Then, the soft Arms were actuated to the predefined pressures, and the linear actuator pulled the cylinder at a fixed velocity (12.5 mm s^−1^ ) until the cylinder separated from the soft gripper. **(B-C)** Vertical load capability of actuation principles II **(B)** and IV **(C)** face different objects. Test object: cylinders with a diameter of 90 mm, 120 mm, and 200 mm and a length of 200 mm. **(D)** Vertical load capability of actuation principles Ⅵ face spheres with a diameter of 90 mm, 120 mm, and 200 mm.


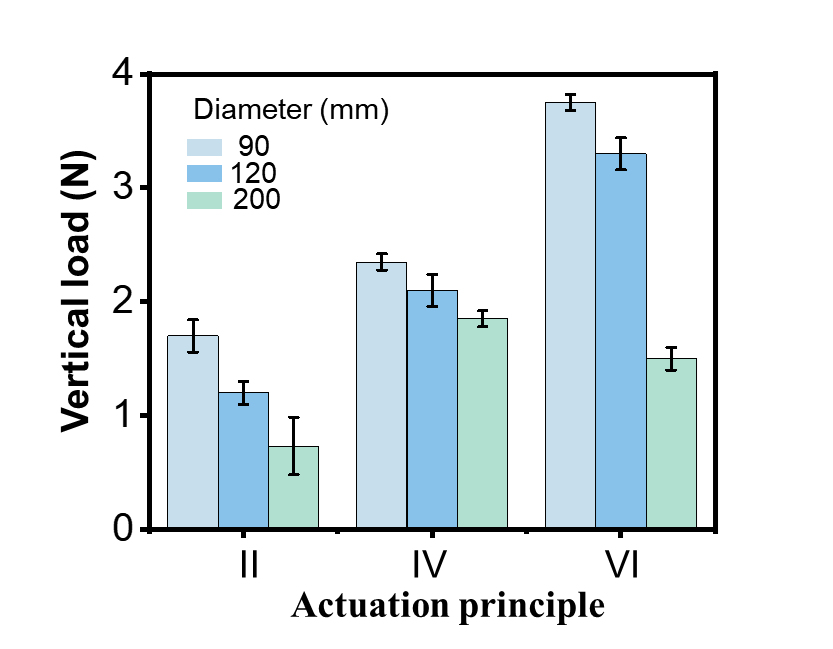


**Fig. S11. The evaluation of the output of the three actuation principles facing different objects.** The grasping force output will increase with more Arms actuated and smaller diameter objects. Error bars show standard deviation from 4 tests.

**
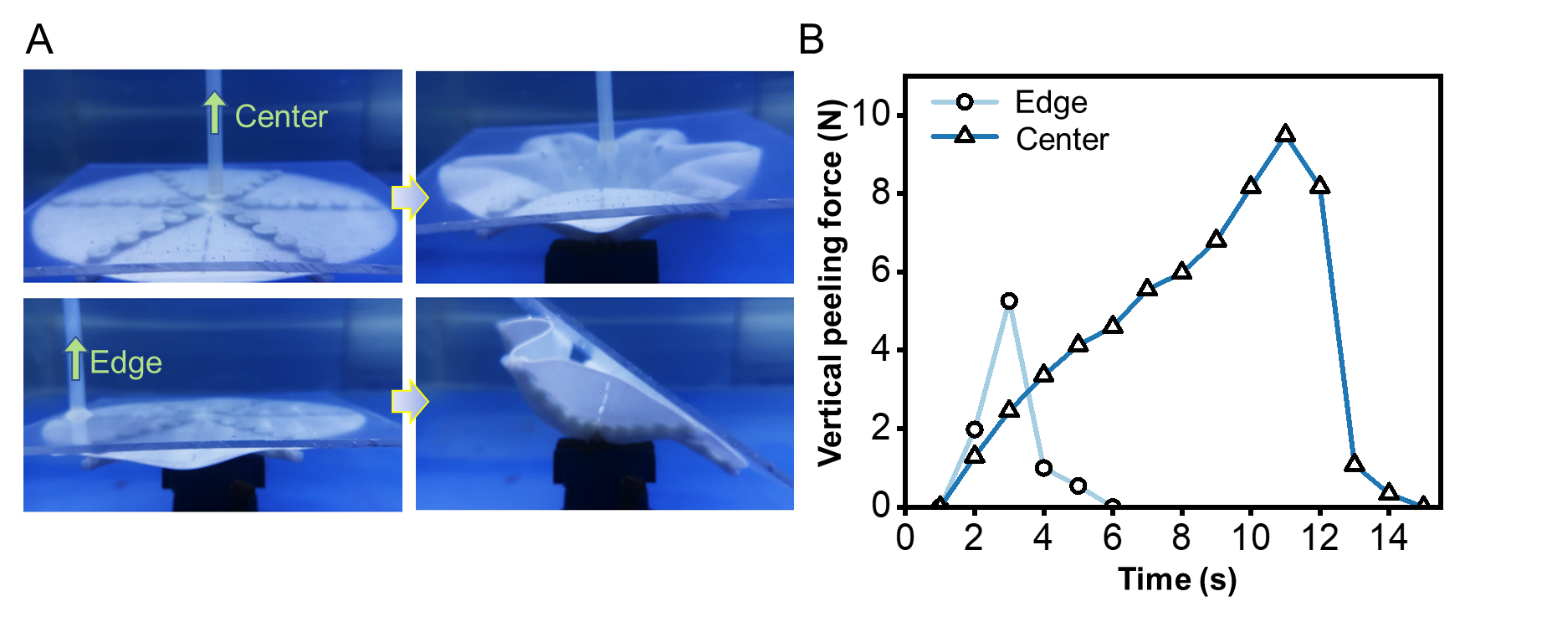
Fig. S12. Suction test in suction mode. (A)** Demonstration of vertical peeling of substrates from the center and edges of the soft gripper. Cover the substrate on the gripper, start and maintain the negative pressure -60 kPa at the same time, then lift the substrate at a speed of 12.5 mm/s until the sucker is entirely detached from the substrate, and record the real-time output in the suction force throughout the process. **(B)** Graph showing the suction when peeling flat substrates vertically from different positions of the soft gripper.


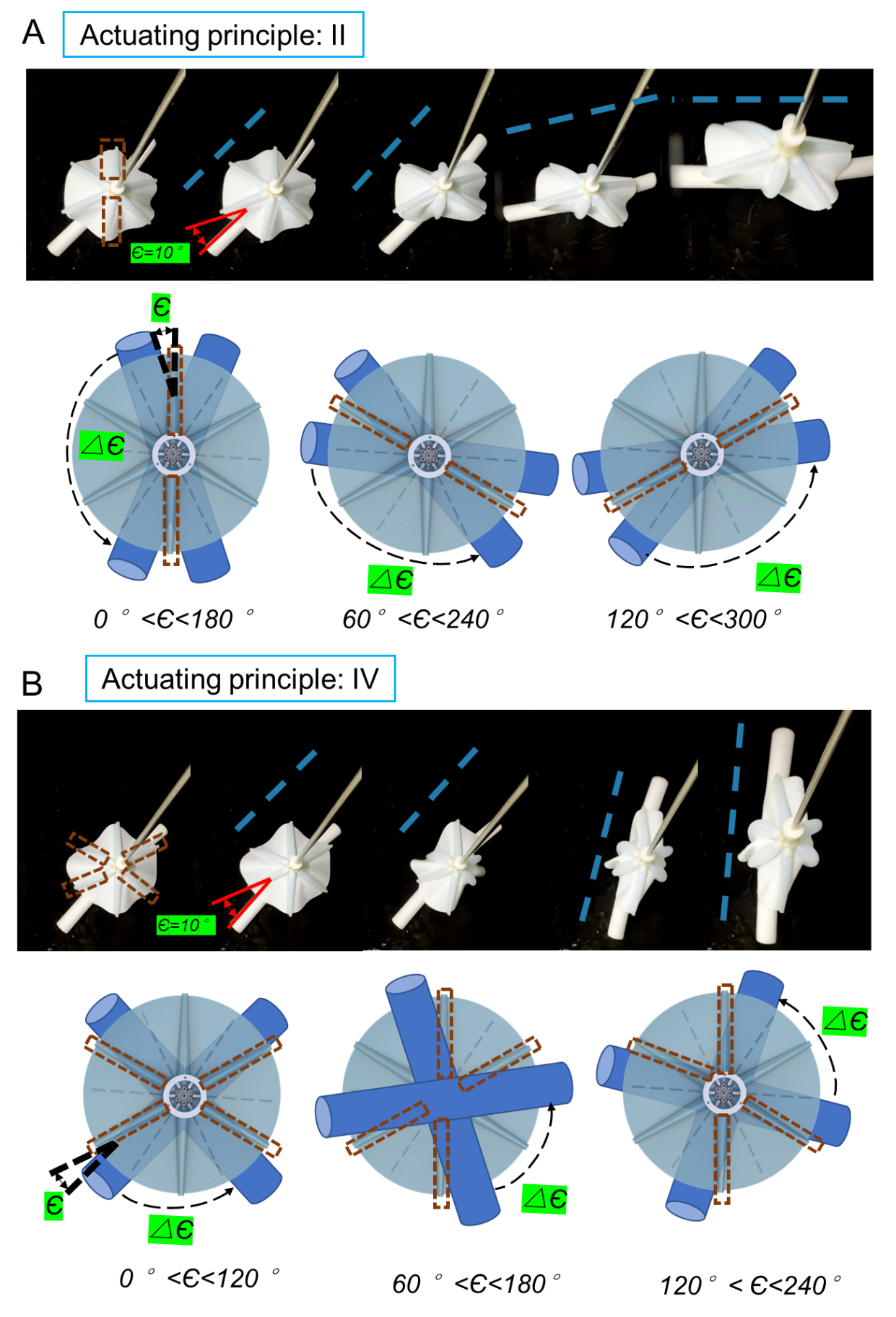


**Fig. S13. Adaptive grasping of objects placed at different angles. (A)** Schematic showing the grasping of the soft gripper under actuation principle II. **(B)** Schematic showing the grasping of the soft gripper under actuation principle Ⅳ. *є* is the angle between the object and the vertical Arm. The dashed line represents the parallel line of the object's position. The gripper can correct the position of objects. The six grasping modes derived from the actuation principle II and Ⅳ can realize the grasping of objects with placement angles of 0°≤*є*≤180°. Due to the symmetrical design of the gripper, objects can be grasped within a 360° placement angle.


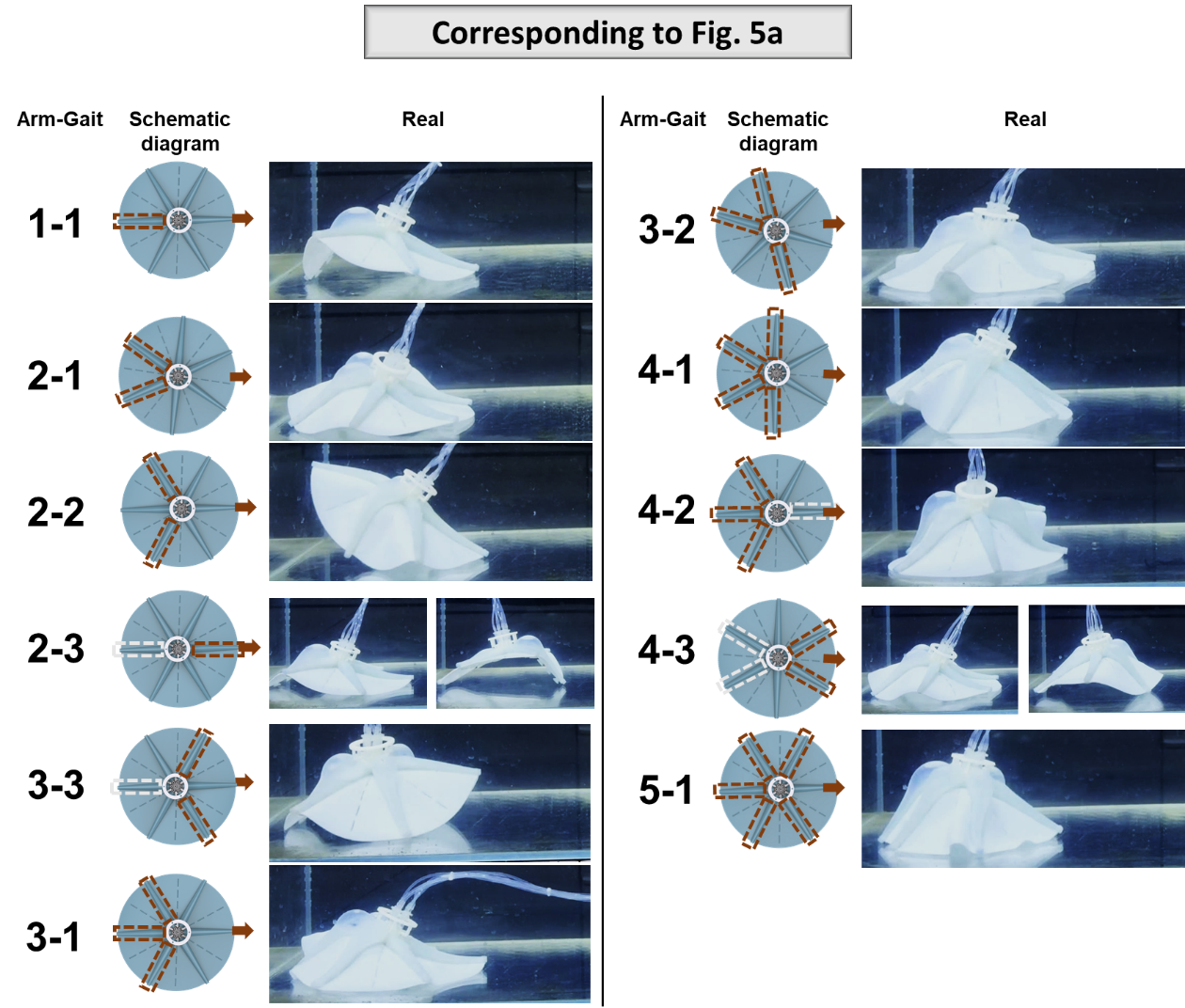


**Fig. S14.** **Schematic showing the gait corresponding to Fig. 5b.** Eleven gaits can be generated by actuating different numbers of Arms. The time required for each gait to bend the Arm during exercise is the same, so the step length measures the speed of movement. So far, gait 2-2 can produce the highest speed relative to other gaits.


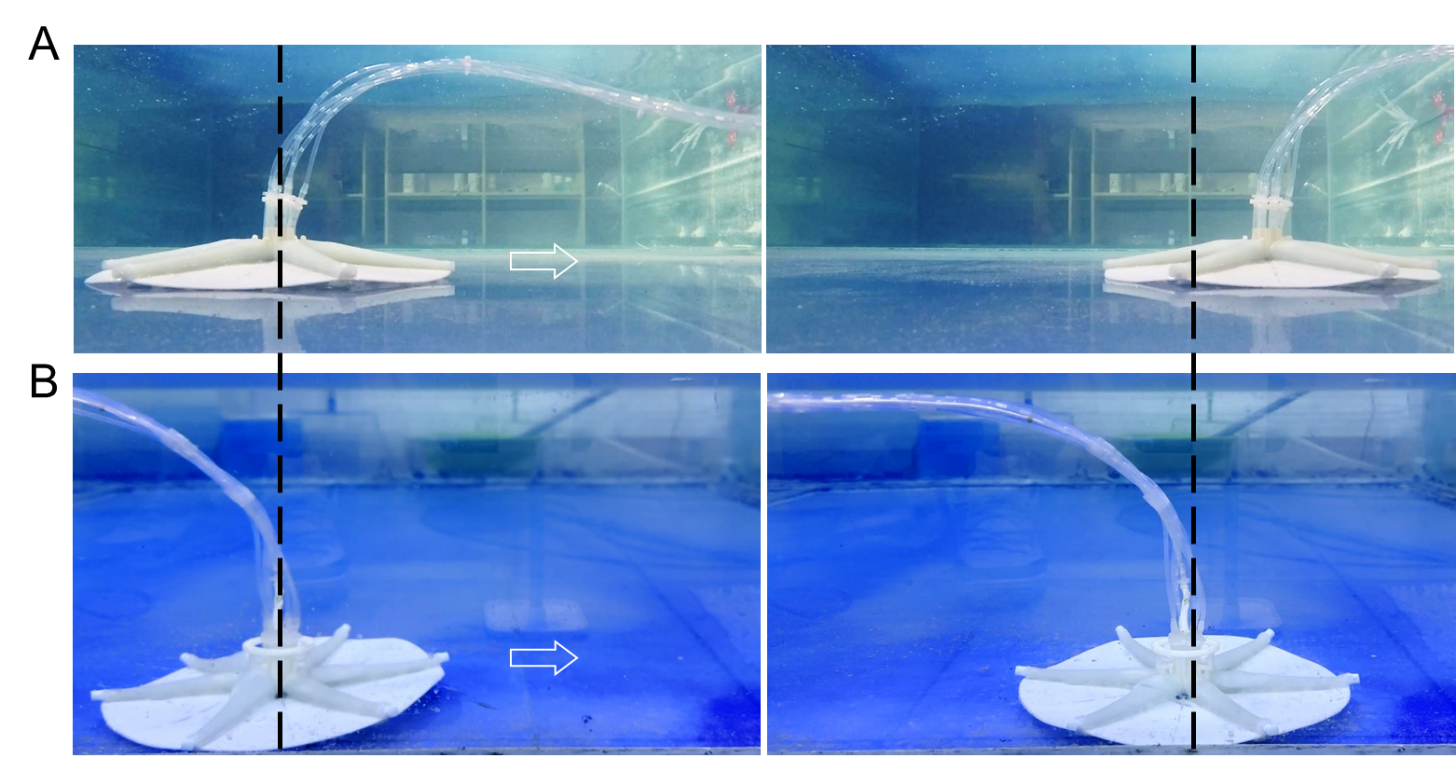


**Fig. S15. Effect of pipe position on gripper movement speed within the same time period.** (**A**). The pipe position is forward in the direction of movement. (**B**). The pipe position is behind the direction of movement.


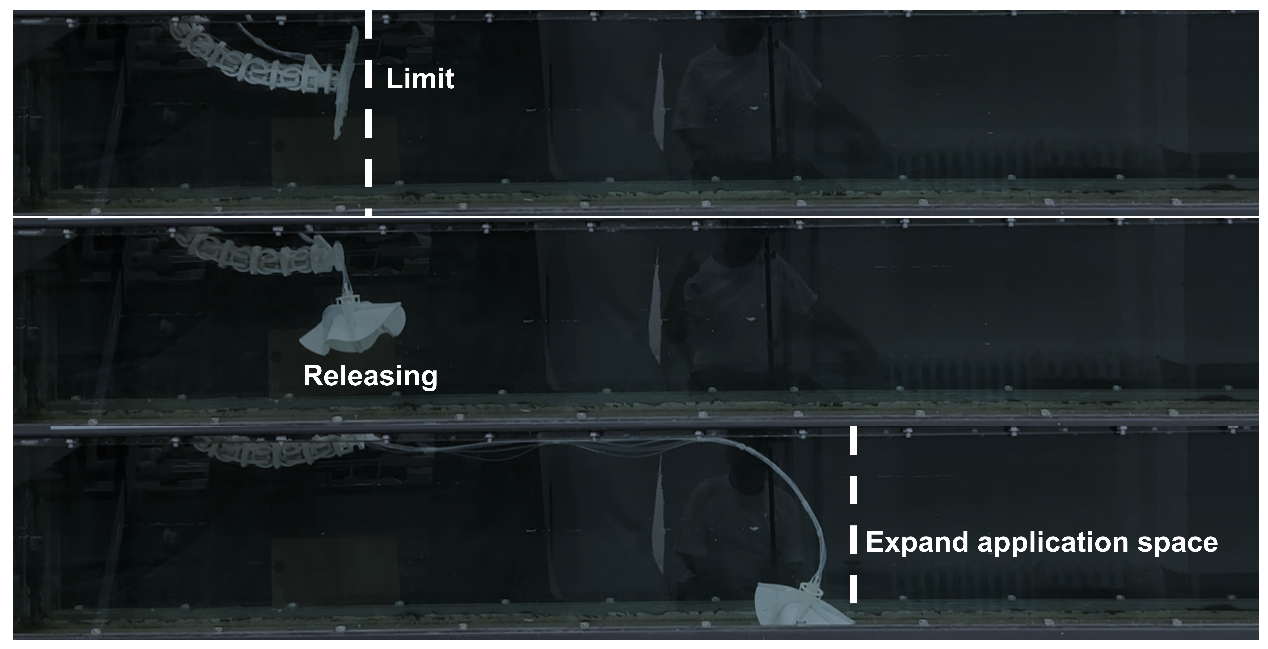


**Fig. S16.** **Demonstration of expanding application space without the limitations of robotic arm.**


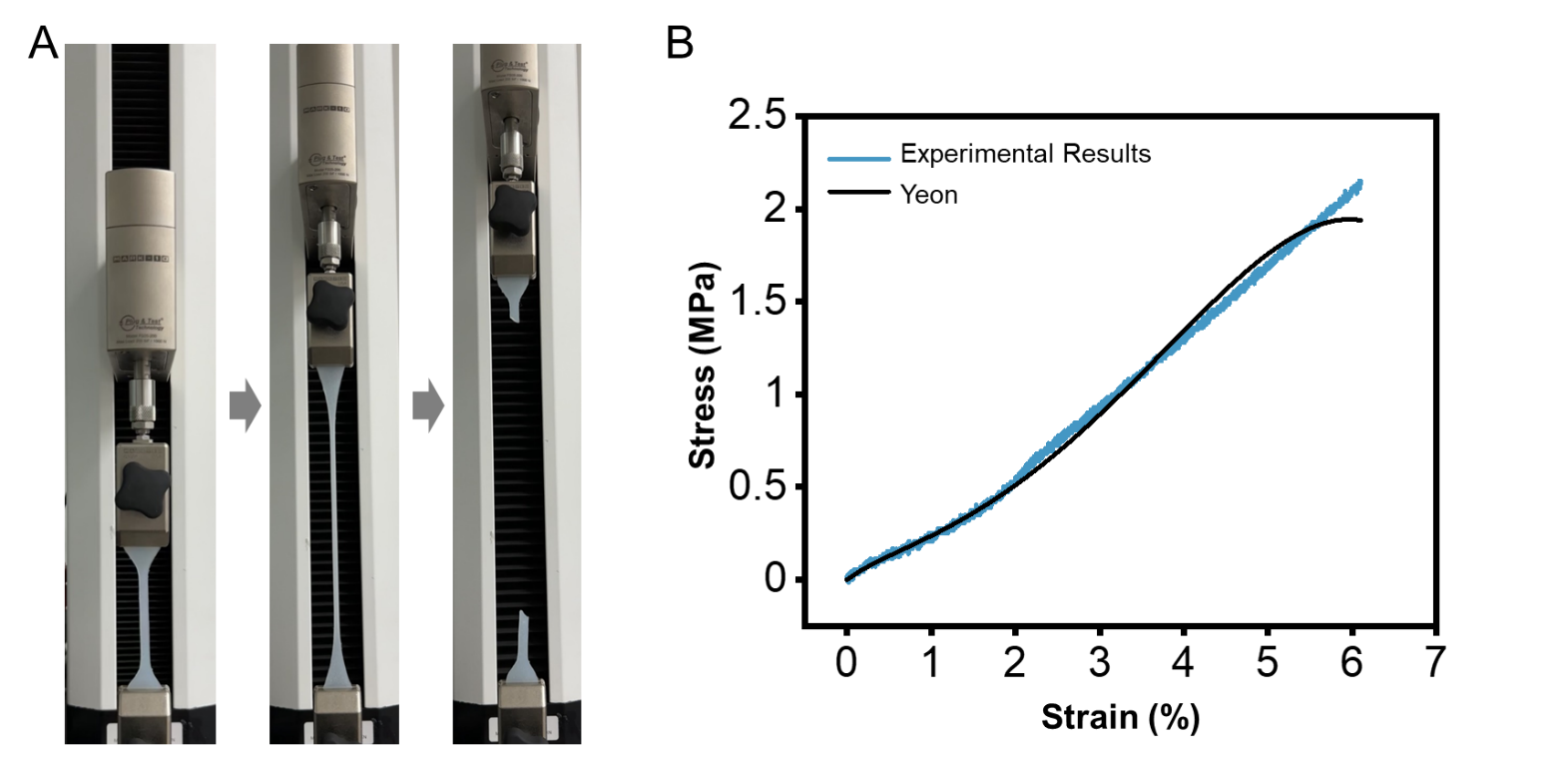


**Fig. S17. Material behavior. (A-B)** Stress-strain curve for Dragon Skin 20 as measured in experiments (continuous line) and predicted using a Yeon hyperelastic material model (dashed line).

To characterize the mechanical response of the silicon rubber used to fabricate our Arms, we tested dog bone-shaped samples (ASTM standard) made out of Dragon skin 20 under uniaxial tension, using a single-axis Instron (Mark-10 F105) with a 100 N load cell. The material behavior up to a strain of 6.2 (i.e., until failure) is reported in Fig. S13. We find that the material response was effectively captured by an incompressible Yeon hyperelastic model (39), whose strain energy is given by：

$$W=\sum_{i=1}^{N} C_{i0}\left( \bar{I}_{1}-3 \right)^{ⅈ}+\sum_{k=1}^{N} \frac{1}{D_{k}}\left( J-1 \right)^{2k}$$

Where J is the volume ratio after deformation to before deformation, for incompressible materials, J=1. $I_{1}$ is the first strain tensor invariant.

$$\bar{I}_{1}=J^{-2/3}$$

C_i0_ and D_k_ are the material parameters. In our model N = 3, C_10_ = 0.06066, C_20_ = 0.00224, C_30_ = -1.8532 * 10^-5^ , and D_1_ = D_2_ = D_3_ = 0 .


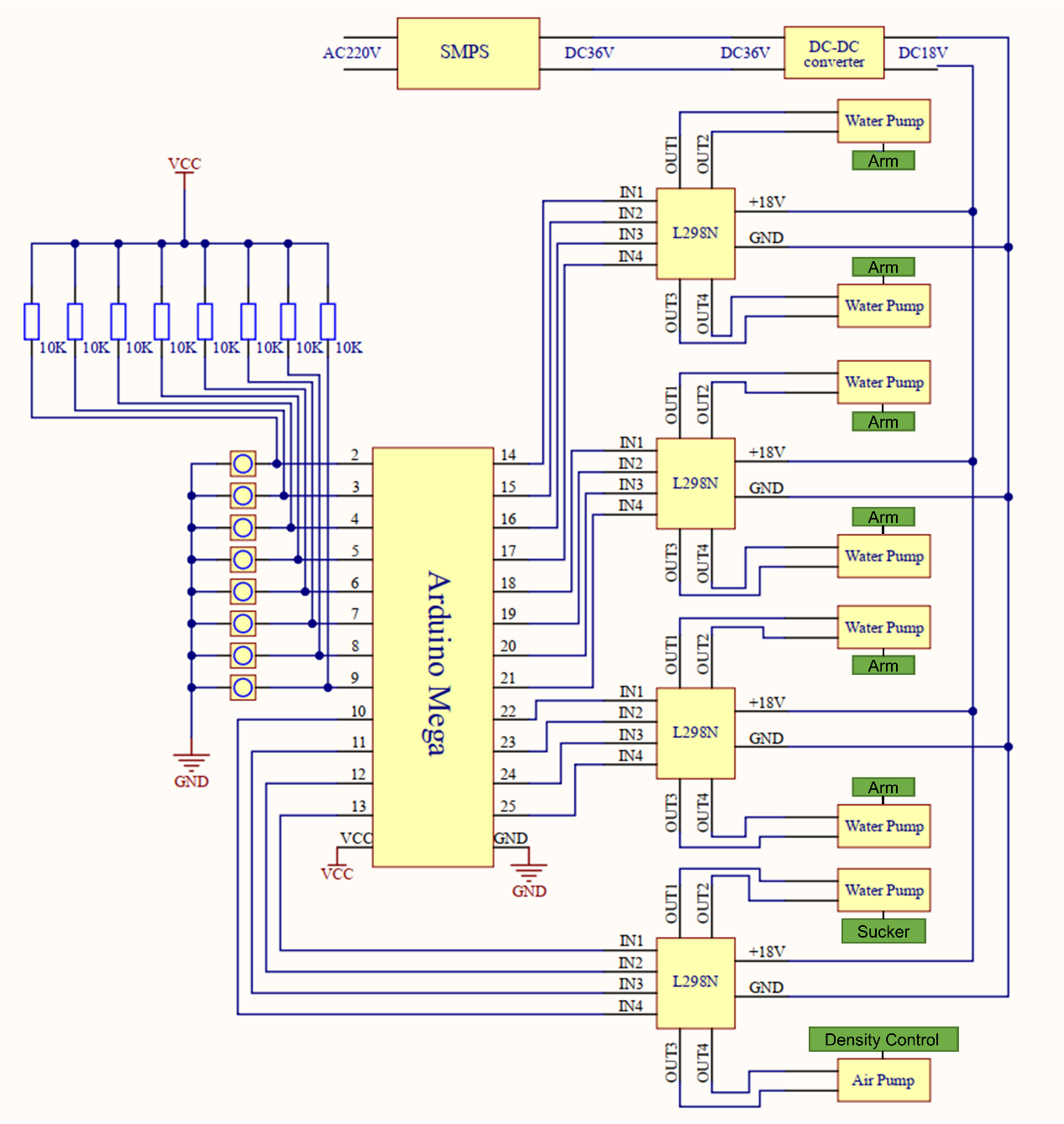


**Fig. S18. Block diagram of the control interface in the soft gripper.** The driving of six Arms is driven by six geared pumps of the same model (GGMN 1200, KOREA), and H-bridge(L298N) modules are used to control positive and negative pressure switching control Arms and suckers (fig. S14). A regulated source powers both Arduino and L298N through a voltage regulation module. Arduino sends the signal to L298N to control the work of each pump. The weight of the overall control system is 1 kg, which is convenient to carry on a small underwater robot.
